# The response and coupling coordination of soil moisture to vegetation in the Yellow River’s primary tributaries: a multi-source data analysis of the Wanchuan River Basin

**DOI:** 10.3389/fpls.2025.1700203

**Published:** 2026-01-19

**Authors:** Xingyu Liu, Xiaodan Li, Xiaoning Zhang, Jiali Yang, Zizhen Li, Haichao Zhang, Xuelu Liu

**Affiliations:** 1 College of Forestry, Gansu Agricultural University, Lanzhou, China; 2 College of Management, Gansu Agricultural University, Lanzhou, China; 3 College of Resources and Environment, Gansu Agricultural University, Lanzhou, China; 4 School of Architecture, South China University of Technology, Guangzhou, China

**Keywords:** coupling coordination, random forest, soil moisture, vegetation dynamics, Wanchuan River Basin, Yellow River tributaries

## Abstract

**Introduction:**

Vegetation status and soil moisture play crucial roles in ecosystem supply- demand services. Understanding the spatiotemporal variations of soil moisture (SM) and vegetation conditions is essential for assessing the stability of terrestrial ecosystems. However, the long-term response mechanisms and synergistic relationships between soil moisture and vegetation in the upper Yellow River’s Gansu section remain unclear, introducing uncertainties in evaluating the Loess Plateau ecosystem.

**Methods:**

Utilizing multi-source data from the Wanchuan River Basin, a major tributary of the Yellow River, an inversion model was constructed to simulate the spatiotemporal distribution of soil moisture at depths of 0–30 cm.

**Results and Discussion:**

The results reveal a distinct spatial pattern of lower moisture in the northern part and higher moisture in the southern part, with no significant variation across soil depths. A comprehensive vegetation index (VEG) integrating net primary productivity (NPP), leaf area index (LAI), and atmosphere-resistant vegetation index (ARVI) was found to be most suitable for this region. Overall vegetation conditions improved annually, with interannual variation following a normal distribution. Significant interaction between vegetation and soil moisture was observed, with the area of statistically significant positive correlation substantially exceeding that of negative correlation, indicating a synergistic relationship between the two variables. Coupling coordination analysis showed notable seasonal differences: in summer, coordination between SM and VEG was significantly divergent (coordinated in the south, uncoordinated in the north), while in other seasons, the relationship was characterized as marginally coordinated. This suggests a need for continuous structural optimization, with marginal coordination representing the predominant state of the soil moisture– vegetation system in the basin.Variable importance screening using the Giniindex- enhanced random forest algorithm identified land surface temperature and potential evapotranspiration as the dominant factors influencing the soil moisture–vegetation coupling coordination degree. These findings provide valuable data and theoretical support for understanding the synergistic mechanisms between soil, water, and vegetation in the Gansu section of the Yellow River, contributing to more effective ecosystem management strategies in the Loess Plateau region.

## 1 Introduction

Ecosystems provide vital services—regulating, provisioning, cultural, and supporting—that sustain human societies (Braat and de Groot, 2012). Among regulatory services, water conservation is critical for watershed stability and erosion control. Its effectiveness largely depends on soil moisture (SM) dynamics, which directly mediate water regulation and ecosystem resilience (Costanza et al., 2017).

Soils constitute the primary reservoir for moisture retention, with SM governing spatial heterogeneity and overall regulatory capacity (Gan et al., 2019). Key soil properties—organic matter, bulk density, and porosity—strongly influence SM retention (Rawls et al., 2003; Yang et al., 2021). Recent advances in remote sensing and modeling have improved SM quantification. Liang et al. (2020) and Liu C. et al. (2022) developed predictive models using multivariate regression and neural networks, while Deodoro et al. (2023) demonstrated SAR-based soil texture inversion. While methods for visualizing SM have matured in recent years, inversion techniques that integrate both soil physicochemical properties and remote sensing indicators still require further investigation. Further integration of remote and ground-based data remains essential to refine these models and enhance ecological management.

Vegetation dynamics significantly influence soil moisture, typically assessed using long-term vegetation parameters such as the Normalized Difference Vegetation Index (NDVI), Enhanced Vegetation Index (EVI), Net Primary Productivity (NPP), and Leaf Area Index (LAI). Hou et al. (2022) utilized NDVI and its coefficient of variation to analyze spatial patterns of grassland degradation/restoration in Qilian County on the northeastern Tibetan Plateau. Similarly, Zhang et al. (2020) applied the CASA model to investigate spatiotemporal changes in grassland NPP in Gansu Province from 1982 to 2011 and examined their relationship with climatic drivers. These findings demonstrate that vegetation assessment based on remote sensing data serves as a powerful tool for visualizing vegetation conditions. Compared to remotely sensed SM estimation, vegetation indicators offer more diverse forms of characterization, with NPP, LAI, and various vegetation indices being commonly used descriptors. However, integrated assessments combining these multiple vegetation indicators remain limited.

The Loess Plateau, located in northwestern China, is a critical region for ecological restoration projects along the mainstem and tributaries of the Yellow River. Accurate evaluation of vegetation conditions and soil moisture directly informs ecological recovery strategies in this arid to semi-arid region. The coupling coordination degree (CCD) model has emerged as an effective quantitative tool for assessing vegetation–soil moisture interactions and their synergistic relationships. Zhao et al. (2022) successfully applied this model to analyze the synergy between soil habitat indicators and vegetation characteristics, establishing its reliability for such evaluations. Building on this, Gao et al. (2022) developed an advanced grey relational coupling model to investigate coupling mechanisms between species composition and soil properties, highlighting the fundamental importance of vegetation pattern–soil moisture interactions in maintaining regional ecological stability.In addition to interactions and synergies between vegetation and soil moisture, external factors affecting both have become a research focus in recent years. Liu et al. (2024) demonstrated through comprehensive analysis that hydrothermal conditions are key factors influencing both vegetation and soil moisture on the Loess Plateau. Zhang et al. (2024) constructed a soil moisture inversion model and integrated it with vegetation indices using the CCD model, identifying topography as the factor with the highest weight among various influencers. Thus, applying the CCD model to couple vegetation and soil moisture provides an effective means to systematically study their interactions and synergistic effects.

Despite its successful application in various ecosystems, research employing the CCD model to examine the synergistic relationship between soil moisture and vegetation dynamics in major Yellow River tributaries of the Loess Plateau remains scarce. Further work is needed to identify the dominant influencing factors and deepen the understanding of their coupled mechanisms. Therefore, this study selects, for the first time, the Wanchuan River Basin—a primary tributary of the Yellow River in the Loess Plateau region of Gansu Province—as the study area. By integrating multi-source parameters including *in-situ* physiological measurements, remote sensing data, and long-term vegetation indices, we established multiple regression models to achieve high-precision spatial visualization of regional soil moisture. Furthermore, using principal component analysis (PCA), we systematically combined multi-source vegetation descriptors—including NPP, LAI, and vegetation indices—to construct a comprehensive vegetation index, enabling large-scale spatial visualization of vegetation conditions. Through high-resolution spatiotemporal correlation analysis and coupling coordination degree modeling, this study provides an in-depth investigation of the interactions and spatial synergies between soil moisture and vegetation characteristics in the Yellow River Basin of the Loess Plateau. In addition, the random forest algorithm was applied to quantify the relative contribution of various influencing factors. These findings offer a theoretical foundation for understanding the sustainable dynamics of soil moisture and vegetation conditions in major tributaries of the Yellow River.

## 2 Materials and methods

### 2.1 Site description

**Figure 1:**
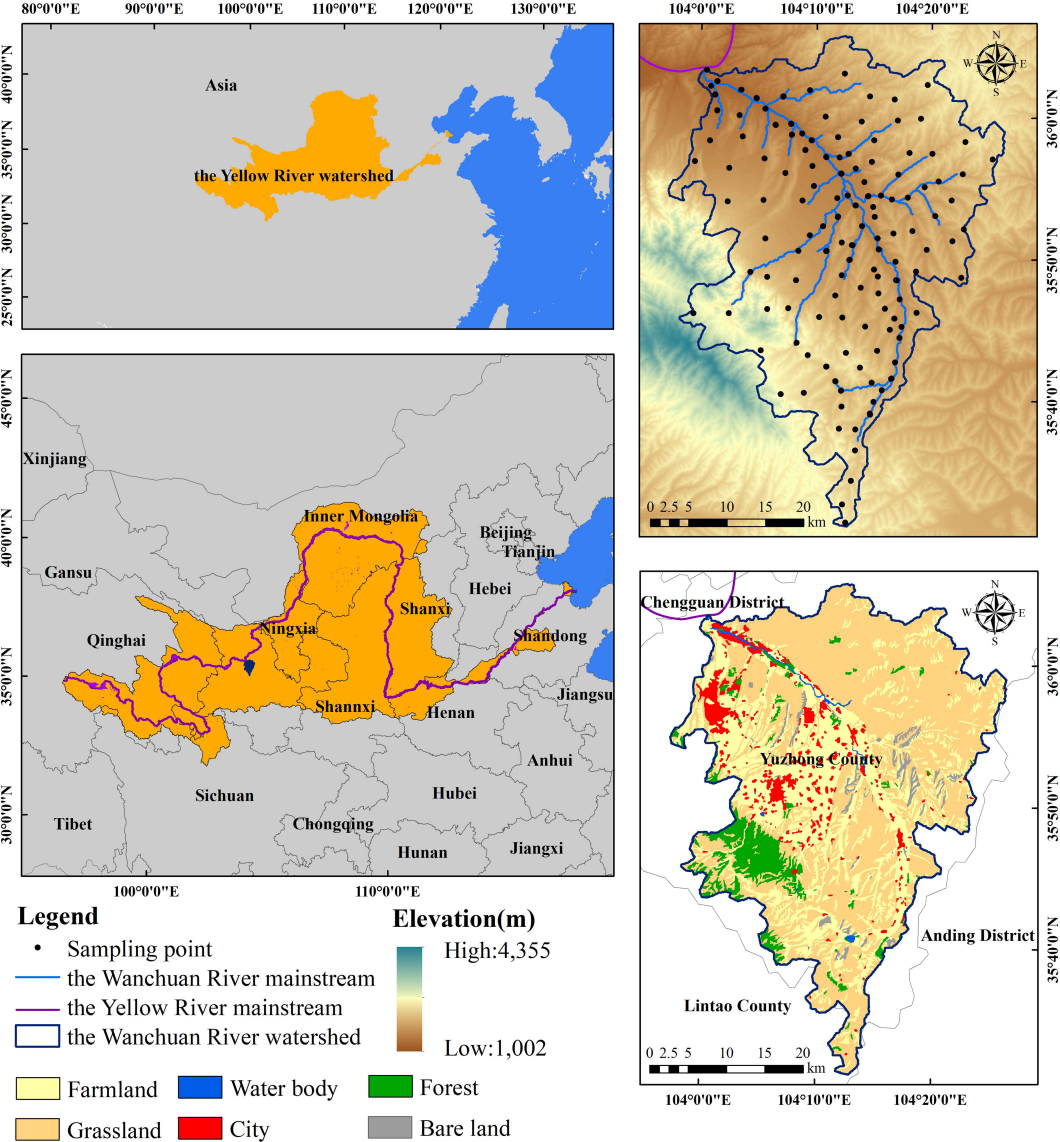
Spatial distribution of study sites.

This study focuses on the Wanchuan River Basin (**Figure 1**), a primary tributary of the upper Yellow River, located in the western Loess Plateau (103°07′-104°02′E, 35°03′-36°01′N). As a crucial ecological barrier for Lanzhou City in Gansu Province, the basin exhibits a stepped topography with higher elevations in the north and south and lower central areas. The main channel spans approximately 80 km, draining a total area of 1,900 km² entirely within Yuzhong County, Lanzhou.

The basin experiences typical temperate continental monsoon climate and holds significant ecological importance. Its headwater originates from the Xinglong Mountain National Nature Reserve (peak elevation: 3,300 m), while its confluence with the Yellow River occurs near Heping Town (minimum elevation: 1,500 m). Topographic gradients create pronounced spatial heterogeneity in vegetation coverage between northern and southern banks. Dominant land use types include cropland and built-up areas, with the Xinglong Mountain area serving as a critical climate regulation zone that maintains regional ecological equilibrium.

### 2.2 Data sources

#### 2.2.1 Soil sample collection and property measurement

**Figure 2:**
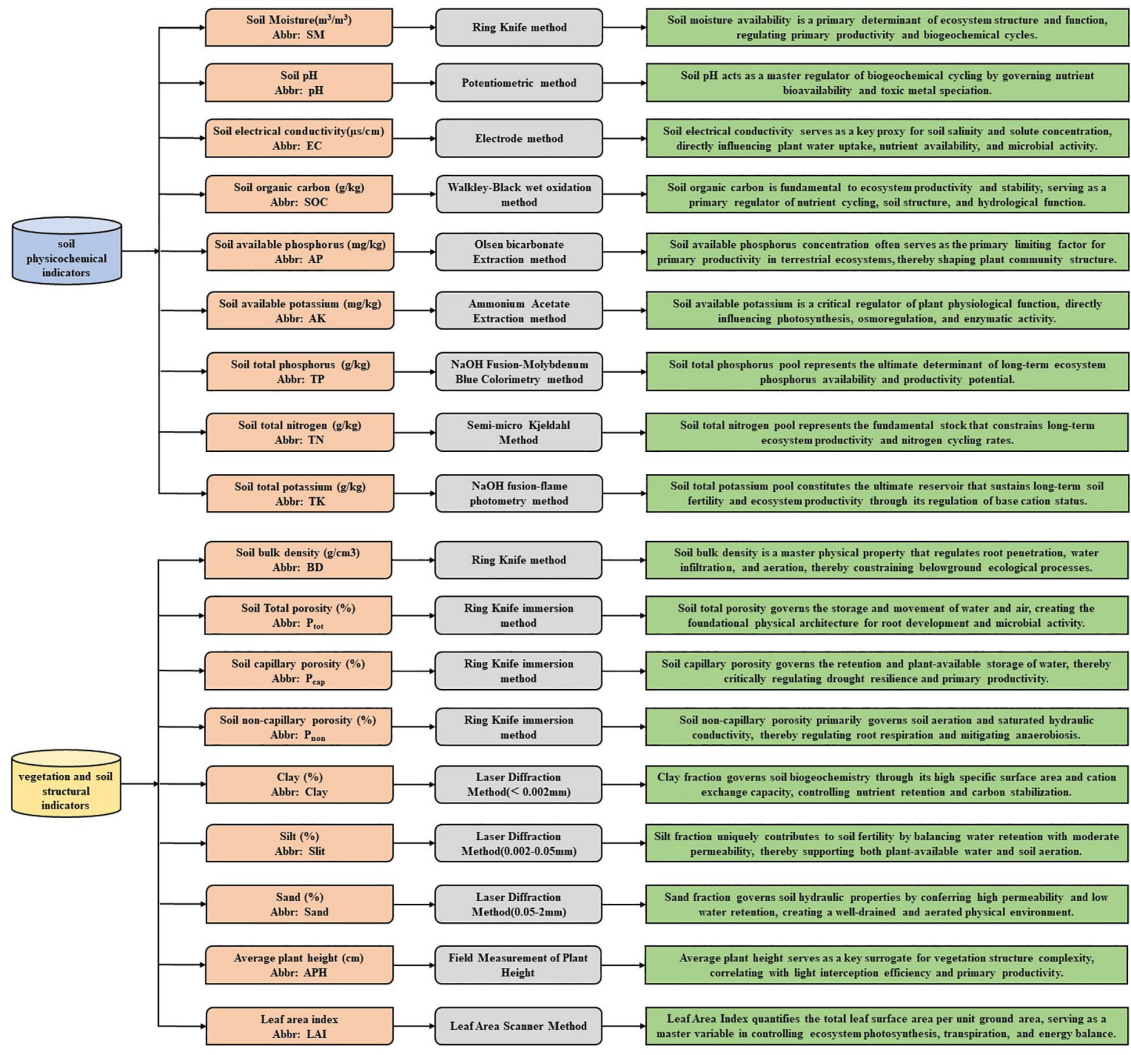
Soil physicochemical indicators: analytical methods and ecological implications.

Field investigations were conducted in the study area from July to August during 2023–2024, employing a stratified random sampling design with 170 sampling points (**Figure 1**), maintaining 1 km intervals between adjacent sites (Yigini and Panagos, 2016). At each sampling location, two 50 cm × 50 cm quadrats spaced 50 m apart were established, with GPS-recorded coordinates and elevation data, vegetation landscape photographs, and measurements of average plant height within quadrats. Soil sampling utilized 100 cm³ cutting rings to collect undisturbed cores for determining fresh weight, porosity, soil moisture and bulk density, while composite samples were obtained via the five-point method across stratified 0–30 cm depths (0–10 cm, 10–20 cm, and 20–30 cm). All samples were air-dried, sieved, and analyzed using a Bettersize2600 laser granulometer for particle size distribution (clay<0.002 mm, silt 0.002–0.05 mm, sand 0.05–2 mm) alongside other physicochemical properties, with detailed analytical methods provided in **Figure 2** (Rosa and Franz, 2005).

#### 2.2.2 Remote sensing data sources and processing

This study employed multi-source remote sensing data to construct models while mitigating multicollinearity effects, primarily utilizing Sentinel-1 and Sentinel-2 datasets from the Copernicus Data Space Ecosystem (CDSE, https://browser.dataspace.copernicus.eu/). Among the selected remote sensing indicators, VH and VV radar bands were obtained from Sentinel-1, representing co-polarized and cross-polarized backscattering coefficients, which are closely related to soil moisture content. The optical bands were acquired from Sentinel-2, comprising 11 spectral bands (B1–B8A and B11–B12, excluding the 60-meter resolution B9 and B10 bands). All bands were uniformly resampled to the same resolution in a GIS platform, followed by coordinate system transformation and watershed mask extraction. The selected vegetation indices were also calculated using Sentinel-2 spectral bands, aiming to incorporate well-performing remote sensing indicators to the greatest extent for constructing the soil moisture regression model.Vegetation indices were systematically derived using the spectral analysis tools within ENVI 5.3 software.To address persistent atmospheric contamination in optical satellite imagery, we implemented a rigorous two-stage quality control protocol. First, temporal outliers caused by cloud and snow cover were identified through a combined radiometric and spectral threshold approach, then reconstructed using linear interpolation algorithms implemented through MATLAB scripting. Second, the corrected time series were processed using Maximum Value Composition (MVC) to generate monthly composite datasets, selectively retaining the highest-quality observations within each temporal window. This integrated methodology significantly reduces atmospheric interference while maintaining the integrity of vegetation spectral signals, following the established practices in remote sensing data processing (Yu et al., 2015). Monthly data were aggregated into seasonal datasets (spring: March-May; summer: June-August; autumn: September-November; winter: December-February) following international seasonal classification standards (Madonsela et al., 2015). The analysis was supplemented with watershed boundary and hydrographic network data provided by Lanzhou Hydrological Station to support comprehensive watershed characterization. The land use data were generated through supervised classification of Sentinel-2 imagery from August 2023, achieving a Kappa coefficient of 0.75. The classification criteria strictly adhered to the Land Use Classification System established by the Chinese Academy of Sciences. All remote sensing data were resampled to a uniform resolution of 20m × 20m, consistent with the spatial resolution of the Sentinel band data.

### 2.3 Development of soil moisture inversion models

This study systematically developed remote sensing inversion models for soil moisture content using multivariate statistical modeling approaches (Lai et al., 2021). Initial Pearson correlation analysis conducted in SPSS 26 identified optimal spectral bands from Sentinel-1/2 data and vegetation indices significantly correlated (*p* < 0.05) with measured soil parameters including moisture content, bulk density, and porosity, which were subsequently selected as modeling variables (Shahriari et al., 2019).

At the same time, the optimal regression model was selected, and the physical and chemical properties of each soil type were characterized using remote sensing bands and vegetation indices, thereby enabling comprehensive spatial and temporal representation of all variables.

Three regression methods were employed: (1) Stepwise Multiple Linear Regression (SMLR) optimized model structure by retaining only significant predictors while mitigating multicollinearity (Khaledian and Miller, 2020; Zare et al., 2019); (2) Partial Least Squares Regression (PLSR) combined principal component analysis with regression to address small-sample, high-dimensional data challenges (Boyan et al., 2015); (3) Ridge regression employs L2 regularization by incorporating a penalty term proportional to the sum of squared weight parameters into the loss function. This approach mitigates overfitting and enhances model generalizability, making it particularly suitable for handling multicollinearity in Sentinel-2 multi-band datasets (Alsaleh et al., 2025). The 170 sampling points were partitioned into 136 modeling samples and 34 validation samples (4:1 ratio) (Madonsela et al., 2015), with model performance evaluated through coefficient of determination (R²), statistical significance (p-value), root mean square error (RMSE), mean absolute error (MAE), and mean absolute percentage error (MAPE) to ensure inversion reliability (Madonsela et al., 2015; Mngadi et al., 2022). The specific calculation equations is as shown in Equations 1–4:

(1) $$R^{2}=\frac{\sum_{i=1}^{n}{\left(x_{i}-\bar{x}\right)}^{2}}{\sum_{i=1}^{n}{\left(x_{2}-\bar{x}\right)}^{2}}$$

(2) $$\;RMSE=\sqrt{\frac{\sum_{i=1}^{n}{\left(x_{1}-x_{2}\right)}^{2}}{n}}$$

(3) $$MAE=\;\frac{1}{n}\sum_{i=1}^{n}\left|x_{1}-x_{2}\right|$$

(4) $$MAPE\;=\;\frac{1}{n}\sum_{i=1}^{n}\left|\frac{x_{1}-x_{2}}{x_{1}}\right|\times 100\%$$

Here, *x_1_* represents the measured value of the soil moisture indicator; *x_2_* denotes the predicted value of the soil moisture indicator; *x̄* indicates the mean value of the measured soil moisture indicators; *n* represents the number of sampling points.

### 2.4 Construction of comprehensive vegetation index

Principal Component Analysis (PCA) is a multivariate statistical method employing dimensionality reduction to transform initial variables into fewer linear combinations that retain most original information. This technique effectively identifies principal components from multiple variables, revealing their quantitative relationships. In this study, PCA was applied to address high correlations among vegetation parameters by using low-dimensional representations to explain the majority of variance in NPP, LAI, and vegetation indices (Zhou et al., 2021). To align with soil moisture measurements from 2017-2024, we first calculated mean values of vegetation indices, LAI, and NPP across their respective raster images. Using ArcGIS’s “Raster to Point” tool, these averaged rasters were converted to vector data, with longitude and latitude fields added to the attribute tables before export, ensuring consistent spatial coordinates and point counts across all three vegetation parameter datasets. Furthermore, seven vegetation indices including NDVI, EVI, Ratio Vegetation Index (RVI), Differential Vegetation Index (DVI), Atmospherically Resistant Vegetation Index (ARVI), Soil Adjusted Vegetation Index (SAVI), and Green Normalized Difference Vegetation Index (GNDVI) were comparatively analyzed through PCA to select the optimal vegetation index, which together with NPP and LAI formed the final composite vegetation index for subsequent analysis. The calculation equation is as follows (Equations 5–11):

(5) $$NDVI=\frac{NIR-Red}{NIR+Red}$$

(6) $$EVI=\frac{2.5\times (NIR-Red)}{NIR+6\times Red-7.5\times Blue+1}$$

(7) $$SAVI=\frac{1.5\times (NIR-Red)}{NIR+Red+0.5}$$

(8) DVI = NIR-Red

(9) $$RVI\;=\;\frac{NIR}{Red}$$

(10) $$ARVI\;=\;\frac{NIR-(2\times Red-Blue)}{NIR+(2\times Red-Blue)}$$

(11) $$GNDVI\;=\;\frac{NIR-Green}{NIR+Green}$$

Among these, NIR refers to the near-infrared band, corresponding to Band 8 (B8) of Sentinel-2 imagery; Red represents the red band, corresponding to Band 4 (B4); Blue denotes the blue band, corresponding to Band 2 (B2); and Green indicates the green band, corresponding to Band 3 (B3).Due to uncertainties in the retrieval accuracy of LAI values derived from leaf area scanners, this study additionally incorporated LAI product data for subsequent analysis. When the retrieval accuracy from scanned data was insufficient, the LAI product data were utilized to support the construction of comprehensive vegetation indices.This study utilized the MOD17A2H and MOD15A2H product datasets from NASA Earthdata Search (https://search.earthdata.nasa.gov/search) to acquire NPP and LAI data. The datasets were resampled to achieve a spatial resolution of 20m × 20m for subsequent analysis. This spatial alignment was implemented to maintain consistency with the resolution characteristics of vegetation indices, ensuring dimensional homogeneity across all remote sensing datasets used for constructing the comprehensive vegetation index (VEG) and performing subsequent correlation analyses.

### 2.5 Partial correlation analysis and significance test of soil moisture and vegetation

The analysis of raster data correlation quantitatively describes the degree of association between raster datasets while outputting correlation coefficients. Significance testing involves formulating hypotheses about the distribution or parameters of random variables, then evaluating these hypotheses against sample data to determine whether observed differences are statistically significant (Gu et al., 2018). Given the well-established relationship between soil moisture and vegetation dynamics, considering the spatiotemporal continuity of our dataset which may not satisfy linearity or normality assumptions, we employed Spearman’s rank correlation analysis to investigate vegetation responses to soil moisture. Using RStudio 4.5.2 with the terra and Hmisc packages, we computed pixel-level Spearman correlation coefficients and associated p-values (derived from t-tests) between soil moisture and vegetation factors at both seasonal and annual scales from 2017 to 2024, thereby capturing their spatiotemporal relationships. The calculation equation is as follows (Equation 12):

(12) $$p=\frac{\sum_{i=1}^{n}\left(R_{VEG,i}-{\bar{R}}_{VEG})(R_{SM,i}-{\bar{R}}_{SM}\right)}{\sqrt{\sum_{i=1}^{n}{\left(R_{VEG,i}-{\bar{R}}_{VEG}\right)}^{2}\sum_{i=1}^{n}{\left(R_{SM,i}-{\bar{R}}_{SM}\right)}^{2}}}$$

where *n* represents the total number of raster cells for both VEG and SM, which must be consistent between the two variables; *R_VEG,i_* ​denotes the rank of the *i*-th VEG value; *R_SM,i_* ​ indicates the rank of the *i*-th SM value; ${\bar{R}}_{VEG}$ is the mean rank of VEG values; ${\bar{R}}_{SM}$ is the mean rank of SM values; and *p* represents the Spearman’s correlation coefficient, which ranges from -1 to 1. To ensure computational validity, all raster datasets were rigorously standardized to identical coordinate systems, resolutions, and dimensional properties (matching row/column counts) prior to analysis.

### 2.6 Construction of a coupling coordination degree model for soil moisture and vegetation

The dynamic characteristics and patterns of soil moisture and the comprehensive vegetation index serve as crucial indicators for assessing soil-vegetation environmental improvements, with their synergistic interactions being quantitatively measurable through coupling degree analysis (Qi et al., 2022). Focusing on these two interconnected systems, we established a coupling degree model for the soil moisture-vegetation system using the following Equation 13:

(13) $$C\;=\;\frac{2\sqrt{VEG\times SM}}{VEG+SM}$$

*VEG* represents the comprehensive vegetation evaluation function, and *SM* denotes the soil moisture evaluation function. The coupling degree *C* between soil moisture and vegetation ranges from 0 to 1. When *C* approaches 1, it indicates a positive coupling state between vegetation and soil moisture, signifying favorable ecological restoration development; when *C* approaches 0, it reflects the opposite condition (Qi et al., 2022).

While *C* effectively quantifies interaction intensity, we advanced the analysis by developing a coupling coordination degree model to holistically evaluate system synergy, incorporating both coupling effects and developmental status to prevent potential misinterpretations from using coupling degree alone (Zuo et al., 2021) (Equations 14, 15):

(14) $$D_{vs}=\sqrt{C\times T}$$

(15) T = α×VEG+β×SM

*D_vs_* represents the coupling coordination degree between soil moisture and vegetation; *T* denotes the comprehensive system harmonization index; *α* and *β* are the undetermined weighting coefficients for the comprehensive vegetation index and soil moisture index respectively, with *α*+*β* = 1. Based on previous research findings, this study considers both factors to be equally important, thus setting *α*=*β*=0.5 (Zuo et al., 2021; Zhang et al., 2024). Similar to *C*, the coupling coordination degree *D_vs_* between soil moisture and vegetation also ranges from 0 to 1. A smaller *D_vs_* value indicates poorer vegetation restoration effectiveness and greater imbalance in the coupling relationship between soil moisture and vegetation.

This study adopted the classification scheme from previous research and divided the coupling coordination degree into five levels using equal intervals (**Table 1**):

**Table 1:** Coupling types between SM and VEG.

Coupling coordination degree	Coupled mode
0 < Dvs ≤ 0.2	Extreme Disorder
0.2 < Dvs ≤ 0.4	Mild Disorder
0.4 < Dvs ≤ 0.6	Barely Coordinated
0.6 < Dvs ≤ 0.8	Moderately Coordinated
0.8 < Dvs ≤ 1.0	Highly Coordinated

### 2.7 Screening of influencing factors for soil moisture-vegetation coupling coordination model based on random forest and Gini index

When assessing the importance of influencing factors in the soil moisture–vegetation coupling coordination model, the random forest algorithm is widely employed. Random forest operates by planting multiple seeds to construct numerous decision trees and aggregating their predictions to enhance model accuracy and robustness. During the construction of each decision tree, selecting the optimal node split point is crucial for its performance. An ideal split should minimize node impurity, with the Gini index serving as a common metric to quantify this impurity. In random forest, the importance of each feature is evaluated by calculating the average reduction in the Gini index achieved when splitting nodes using that feature. The relative importance of a feature is then determined by the proportion of its average Gini index reduction to the total reduction across all features (Breiman, 2001; Louppe, 2014; Liu et al., 2025). The calculation equation is as follows (Equation 16):

(16) $$\;Gini\left(m\right)=\;1-\sum_{j=1}^{k}{\left[p\left(j│m\right)\right]}^{2}$$

*p (j | m)* represents the probability of class *m* at node *j*. When the feature *X_k_* is used for node splitting, the change in the Gini index before and after the split is calculated. The calculation equation is as follows (Equation 17):

(17) $$VIM_{k}\;=\;Gini\left(m\right)-Gini\left(r\right)-Gini\left(l\right)$$

*Gini(m)* denotes the Gini index of node *m* before splitting, while *Gini(r)* and *Gini(l)* represent the Gini indices of the right and left child nodes after splitting, respectively.

Within a single decision tree, the Gini changes from all split points that use feature X_k_ are accumulated. Across the entire random forest, the total Gini change for feature X_k_ is computed by summing its Gini changes over all decision trees. The calculation equation is as follows (Equation 18):

(18) $$VIM_{Gini,k}\;=\;\sum_{i=1}^{n}VIM_{ik}$$

*n* represents the total number of trees in the random forest.

The importance score of a feature is obtained by calculating the percentage of its total Gini change relative to the total Gini changes of all features. The calculation equation is as follows (Equation 19):

(19) $$VIM_{k}\;=\;\frac{VIM_{k}}{\sum_{i=1}^{c}VIM_{i}}$$

*c* represents the total number of features.

The influencing factors selected in this study for the soil moisture–vegetation coupling coordination degree are categorized into anthropogenic activity factors (population density), topographic factors (elevation, slope, aspect), climatic factors (mean annual temperature, mean annual precipitation, land surface temperature, and potential evapotranspiration), covering all direct driving elements. Among them, the population density dataset was developed by the Oak Ridge National Laboratory (ORNL) of the U.S. Department of Energy and provided by East View Cartographic (https://landscan.ornl.gov/). The mean annual temperature, mean annual precipitation, and potential evapotranspiration (Peng, 2022) datasets were obtained from the National Tibetan Plateau Data Center (https://data.tpdc.ac.cn/zh-hans/data/). The land surface temperature dataset (Wan et al., 2021) was sourced from the NASA Earthdata portal (https://www.earthdata.nasa.gov/). Topographic factors were derived from DEM data (https://search.earthdata.nasa.gov/search) using the spatial analysis tools in ArcGIS 10.7. All datasets were resampled to a uniform resolution of 20m × 20m to maintain consistency with the data described in Sections 2.2.2 and 2.4.

## 3 Results

### 3.1 Screening of soil moisture indicators

Analysis of three soil depths revealed consistent correlations between soil moisture (SM) and six physicochemical parameters: bulk density (BD), capillary porosity (P_cap_), non-capillary porosity (P_non_), available potassium (AK), total nitrogen (TN), and total phosphorus (TP). Among these, BD and porosity measures (P_cap_ and P_non_) showed the strongest correlations (**Supplementary Figure S1**).

Remote sensing data identified four significant reflectance bands (VH, B12, B8, and B8A) that maintained stable relationships with SM across all soil depths. Soil texture analysis demonstrated that clay content significantly affected SM only in surface soils (0–10 cm depth), revealing depth-dependent textural control on moisture retention. Since the LAI obtained from the leaf area scanner showed no significant correlation with any of the factors considered, it could not be effectively used for inversion or spatial representation. Therefore, LAI product data were employed for the subsequent stages of this study.

### 3.2 Evaluate the fitting results of the model

This study investigated the relationship between soil characteristics and soil moisture through regression modeling using statistically significant soil physicochemical indicators and reflectance bands strongly correlated with soil moisture (**Supplementary Figure S1**). Although sand content (0–10 cm depth) and leaf area index (20–30 cm depth) showed significant correlations with soil moisture, they were excluded from regression models due to the absence of significantly correlated spectral bands required for reliable predictive modeling.

The developed soil moisture regression model demonstrated excellent predictive performance across all three soil depths, with R² values exceeding 0.95, indicating strong agreement between predicted and observed values. Error analysis further validated the model’s accuracy, revealing that except for ridge regression at 0–10 cm and SMLR at 10–20 cm depth which exhibited relatively higher MAPE, all other RMSE, MAPE and MAE values remained within acceptable ranges, collectively confirming the model’s high precision in soil moisture estimation.

**Figure 3:**
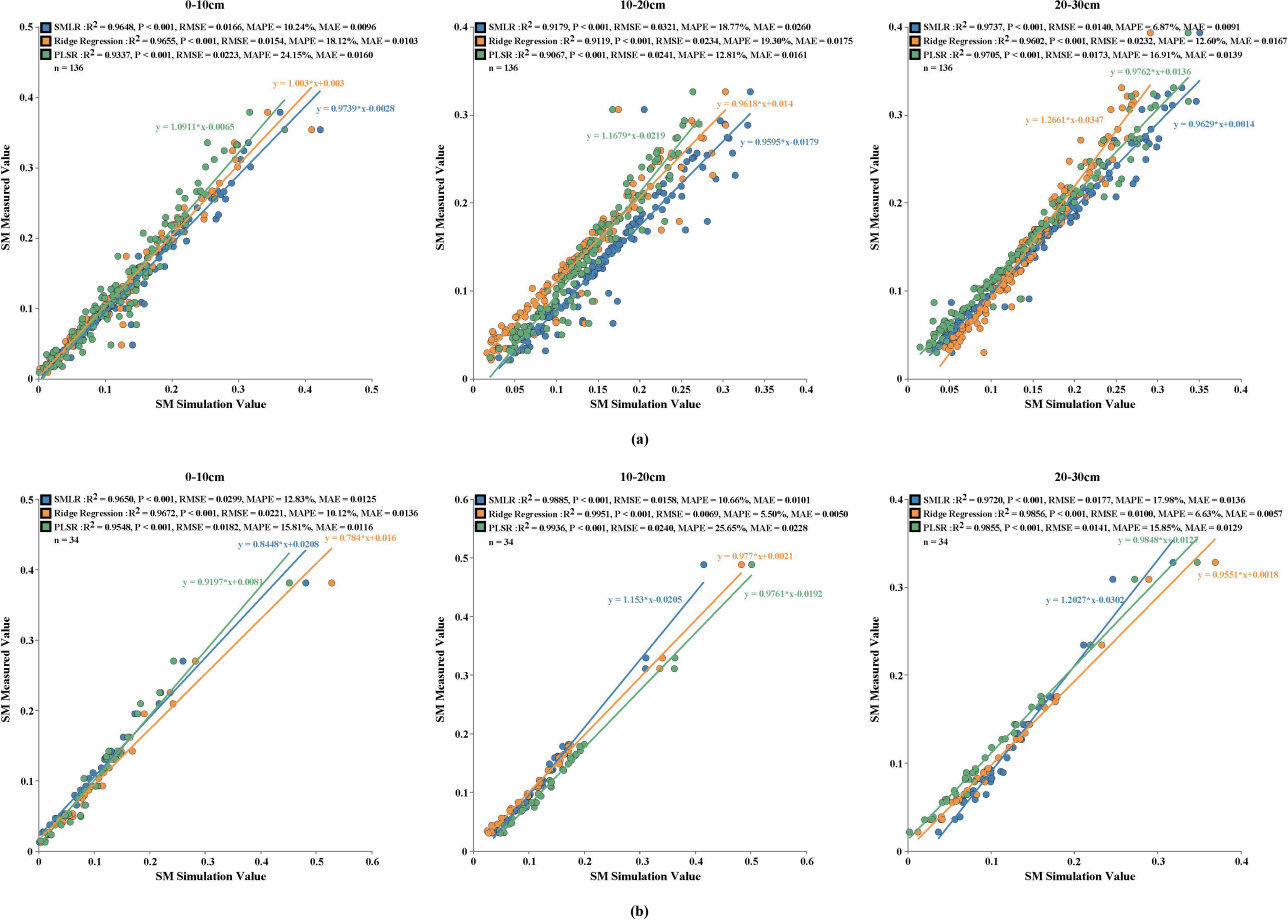
Regression equations for soil moisture at different depths: **(a)** Training set; **(b)** Validation set.

Among the three evaluated regression methods (all with *p* < 0.001), model selection was conducted based on R² values while maintaining RMSE<0.05, MAE<0.02, and MAPE between 0-20%. For 0–10 cm depth, while ridge regression achieved the highest R², SMLR showed superior performance in error metrics and was deemed more suitable. At 10–20 cm depth, ridge regression performed best with the highest R² values and lowest RMSE. For 20–30 cm depth, SMLR outperformed other methods across all evaluation metrics, establishing itself as the optimal choice. This systematic model selection process effectively addressed multicollinearity challenges in soil moisture inversion studies (**Figure 3b**). The final regression equations are presented in **Table 2**, with correlation analysis in **Supplementary Figure S1** indicating that BD and P_cap_ had the greatest influence on model construction. Importantly, the ridge regression equation for 10–20 cm depth demonstrated the necessity of integrating multi-source data for inversion model development, which yields more accurate results.

**Table 2:** Optimal equations for soil moisture function index at various depths.

The optimal regression equation	Equation type
SM(0-10cm) = -0.105 + 0.013 * P_cap_ + 0.084*BD	SMLR
SM(10-20cm) = -0.123 + 0.012 * P_cap_ + 0.101 * BD + 0.002 * TP + 0.016 * TK + 0.000004*B12-0.00001*B6	Ridge Regression
SM(20-30cm) = -0.115 + 0.013* P_cap_ +0.091* BD	SMLR

### 3.3 Spatio-temporal dynamic analysis of soil moisture content

#### 3.3.1 Seasonal scale spatial variation characteristics

The regression equations in **Table 2** were used to estimate soil moisture content by combining soil physicochemical indicators with remote sensing bands and vegetation indices. Analysis of seasonal average spatial distribution patterns across different soil depths (2017-2024, **Supplementary Figure S2**) revealed several key findings:

During summer months, all soil layers showed similar spatial distributions, with high moisture values concentrated in the southern bank and Xinglong Mountain areas, while low values predominated in northern regions, creating a distinct south-to-north moisture gradient. In other seasons (spring, autumn, and winter), surface (0–10 cm) and deep layers (20–30 cm) display remarkably similar distribution patterns, though with significant moisture content differences between them. In contrast, the middle layer (10–20 cm) exhibited relatively uniform moisture distribution with minimal spatial variation.

These results demonstrate that while seasonal moisture patterns show some consistent features across depths, surface and deep layers display pronounced spatial heterogeneity throughout the year, contrasting sharply with the more stable middle layer (10–20 cm). This differential behavior indicates that moisture in both surface (0–10 cm) and deep layers (20–30 cm) is less stable and more variable, while the intermediate 10–20 cm layer maintains greater stability with consistent spatial patterns across the entire watershed.

#### 3.3.2 Annual scale spatial variation characteristics

**Figure 4:**
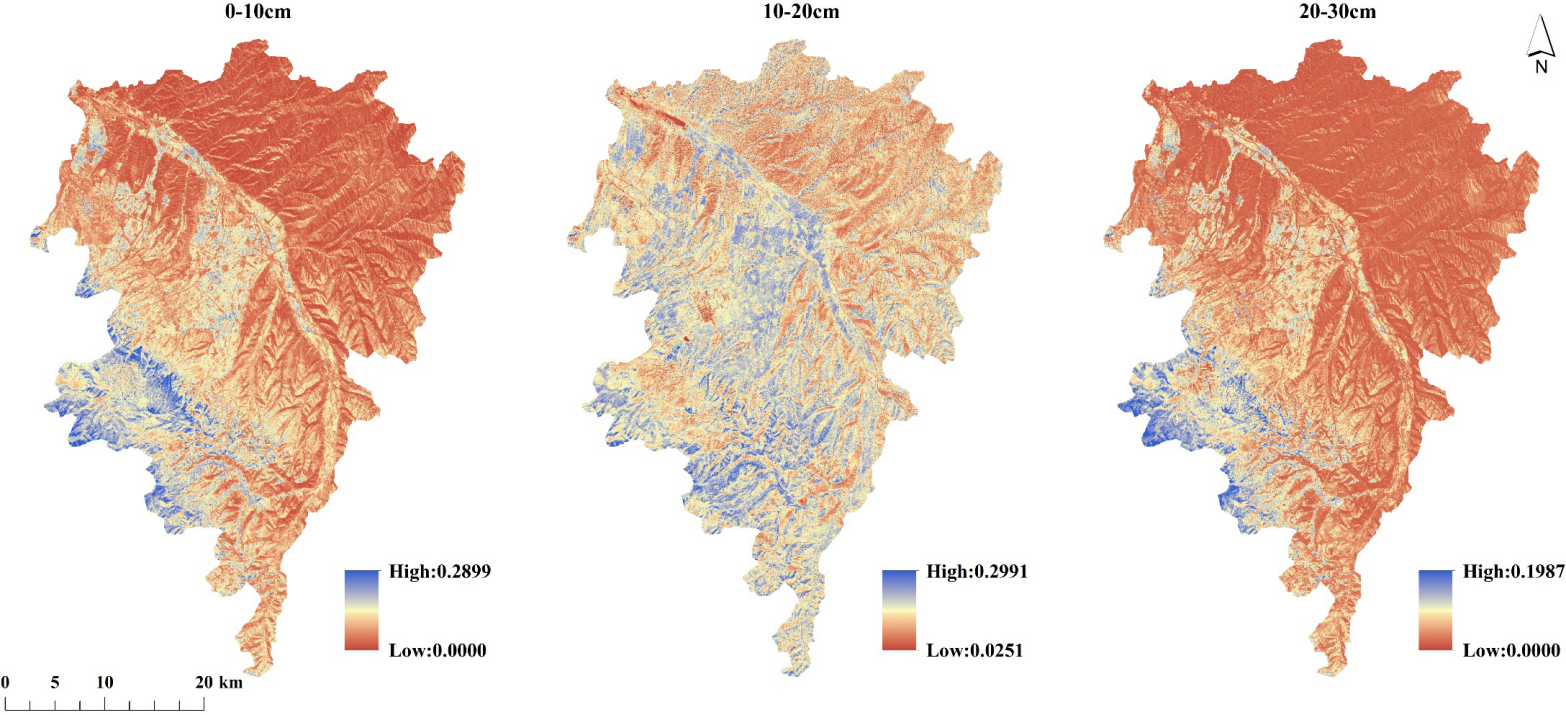
The annual-scale spatial distribution of SM (2017–2024).

Analysis of annual average SM spatial distribution (**Figure 4**) revealed distinct depth-dependent patterns. Compared to intermediate depths, areas with low moisture content covered more extensive spatial ranges in both surface and subsurface layers, primarily distributed in northern and eastern regions of the study area. The Xinglong Mountain area showed unique vertical SM distribution characteristics, maintaining consistently higher moisture in surface and deep layers while exhibiting intermittently lower moisture at intermediate depths.

### 3.4 Construction of Integrated Vegetation Index

#### 3.4.1 Correlation matrix analysis

The annual average rasters of the seven vegetation factors, along with NPP and LAI, were converted to point data using the Raster to Point tool in ArcGIS. This ensured consistency in spatial boundaries and resolution. The exported data were then analyzed using Principal Component Analysis (PCA) in SPSS 26. **Supplementary Table S1** presents the Pearson correlation coefficients between each vegetation index and both NPP and LAI. The results demonstrate that only ARVI and NDVI exhibited correlations with LAI exceeding 0.6 (*p* < 0.05 for all significant relationships).

#### 3.4.2 KMO and bartlett tests

The Kaiser-Meyer-Olkin (KMO) measure effectively reflects partial correlations among variables, with values approaching 1 indicating optimal conditions for principal component analysis (PCA), while values below 0.5 suggest unsuitable conditions for factor analysis. As presented in **Supplementary Tables S2a-g**, ARVI demonstrated a KMO value of 0.652 (≈0.7), significantly exceeding the 0.50 threshold and confirming its suitability for PCA. Notably, none of the other vegetation indices achieved KMO values above 0.6. Bartlett’s Test of Sphericity yielded statistically significant results (*p* = 0.000< 0.05), verifying substantial correlations among variables that were consistent with the correlation matrix results (**Supplementary Table S1**). Based on these robust diagnostic tests, ARVI was selected in combination with NPP and LAI for constructing the integrated vegetation index, as these parameters collectively exhibited superior sampling adequacy (KMO > 0.65) and statistically verified inter-correlations (*p* < 0.001). This selection process ensured optimal dimensionality reduction while preserving the most hydrologically significant vegetation characteristics for subsequent analyses.

#### 3.4.3 Comprehensive evaluation of vegetation based on principal component analysis

The communality analysis of original variables revealed exceptionally high variance extraction values for the selected parameters, with NPP, LAI, and ARVI demonstrating extraction values of 0.927, 0.935, and 0.999 respectively. These results indicate that the principal components explain 92.7%, 93.5%, and 99.9% of the variance in LAI, NPP, and ARVI, confirming their outstanding suitability for dimensionality reduction (**Table 3**).

**Table 3:** Table of common factor variances for vegetation factors.

Vegetation factors	Initial value	Extract value
Zscore: NPP	1.000	0.927
Zscore: LAI	1.000	0.935
Zscore: ARVI	1.000	0.999

The principal component analysis yielded significant dimensionality reduction results, with only the first component demonstrating an initial eigenvalue greater than 1 (2.322), accounting for 77.408% of the total variance in the dataset - surpassing the conventional 70% threshold for adequate representation. Notably, while the second component’s eigenvalue (0.539) fell below the Kaiser criterion threshold, its inclusion substantially improved the cumulative variance explanation to 95.376%. This two-component solution therefore captures nearly all (95.4%) of the systematic variation in the original vegetation parameters, representing an 18.0% improvement in explanatory power over the single-component model (**Table 4**).

**Table 4:** Total variance explained for vegetation factors.

Component	Initial eigenvalue			Extraction sums of squared loadings			Rotation sums of squared loadings		
	Total	Percentage of variance	Cumulative%	Total	Percentage of variance	Cumulative%	Total	Percentage of variance	Cumulative%
1	2.322	77.408	77.408	2.322	72.408	77.408	1.778	59.276	59.276
2	0.539	17.968	95.376	0.539	17.968	95.376	1.083	36.099	95.376
3	0.139	4.624	100.000						

**Figure 5:**
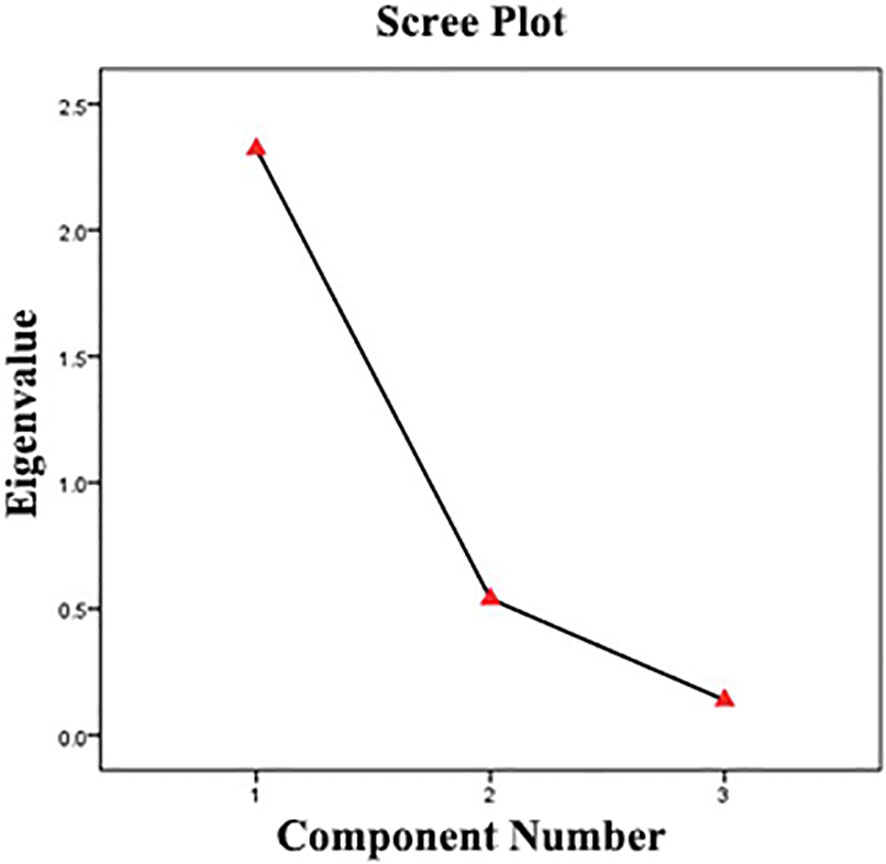
Scree plot.

Furthermore, the scree plot analysis revealed a distinct inflection point occurring at the second principal component, providing additional statistical justification for retaining two components in our model (**Figure 5**).

Based on the total variance explanation (**Table 4**) and component matrix (**Table 5**) results, the final component score coefficient matrix (**Table 6**) yields the following principal component equations:

**Table 5:** Component matrix for vegetation factors.

Vegetation factors	Component	
	1	2
Zscore: NPP	0.935	-0.228
Zscore: LAI	0.919	-0.300
Zscore: ARVI	0.776	0.630

**Table 6:** Component score coefficient matrix for vegetation factors.

Vegetation factors	Component	
	1	2
Zscore: NPP	0.570	-0.130
Zscore: LAI	0.637	-0.245
Zscore: ARVI	-0.397	1.159

F_1_ = 0.570×NPP + 0.637×LAI - 0.367×ARVI
F_2_ = -0.130×NPP - 0.245×LAI + 1.159×ARVI

The absolute values of coefficients reveal distinct ecological interpretations:

1. F_1_, LAI demonstrates dominant influence (0.637), followed by NPP (0.570) and ARVI (-0.367), suggesting this component primarily represents vegetation structural characteristics.
2. F_2_ shows ARVI’s overwhelming contribution (1.159), indicating spectral reflectance dominance in this component.

The composite score weights were derived from each component’s proportion of total eigenvalues, with rotated sums of squared loadings being 1.778 (F_1_) and 1.083 (F_2_). This produces the integrated formula:

F = (1.778×F_1_ + 1.083×F_2_)/(1.778 + 1.083) = 0.594×F_1_ + 0.406×F_2_

Through coefficient normalization of vegetation indicators, the final Comprehensive vegetation index (VEG) is calculated as:

VEG = 0.372×NPP + 0.370×LAI + 0.258×ARVI

#### 3.4.4 Spatio-temporal distribution characteristics of the comprehensive vegetation index

**Figure 6:**
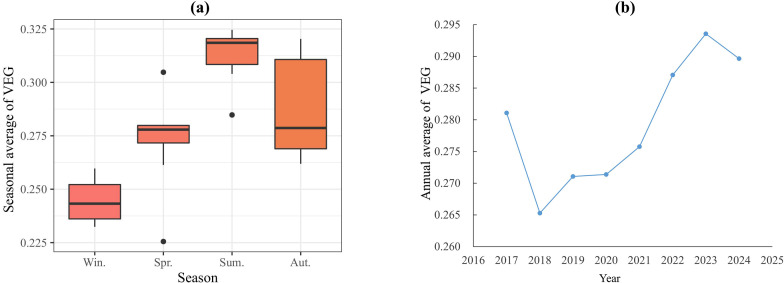
Mean of VEG from 2017 to 2024: **(a)** seasonal scale; **(b)** annual scale.

The temporal and spatial analysis of the Comprehensive vegetation index (VEG) revealed distinct patterns across multiple scales. Seasonal boxplots consistently demonstrated peak VEG values during summer months, with the distribution characteristics approximating normal distribution across all temporal scales (**Figure 6**). Annual trend analysis showed sustained vegetation improvement from 2018 onward, with only a slight decline observed in 2024, suggesting generally positive vegetation dynamics in the study area.

**Figure 7:**
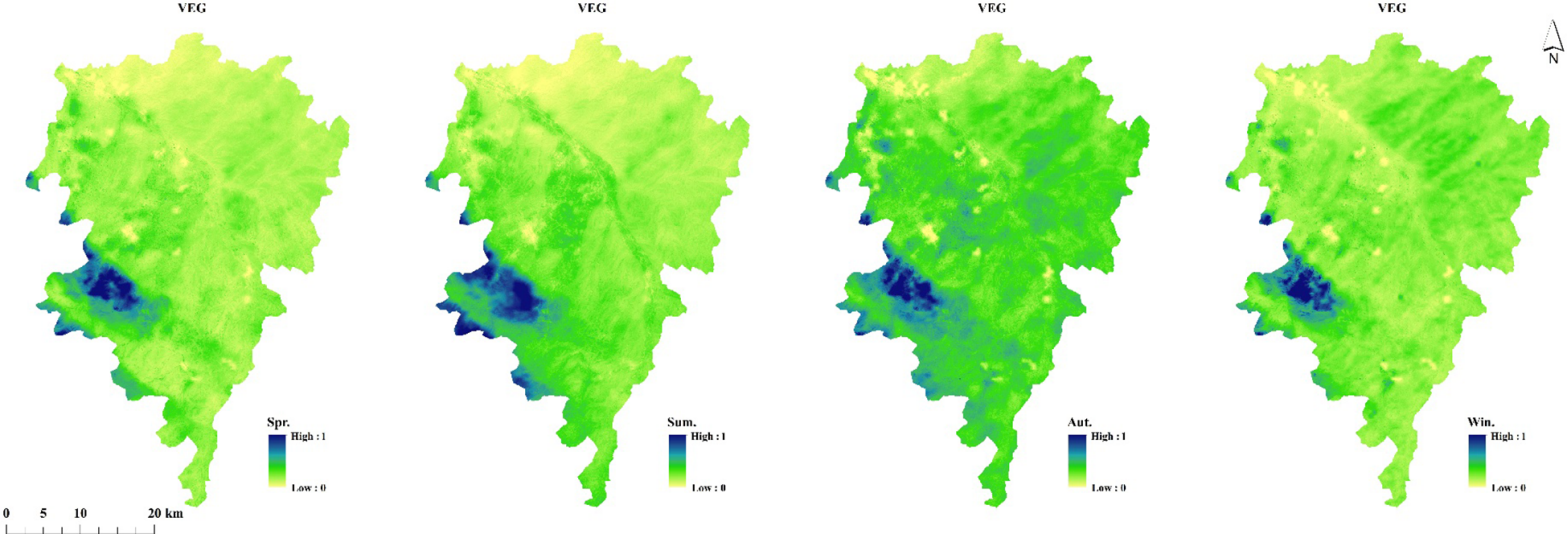
The seasonal-scale spatial distribution of VEG from 2017 to 2024.

Spatially, the southwestern Xinglong Mountain area consistently exhibited the highest VEG values across all seasons, a pattern strongly correlated with the established forest park in this region. Notably, the spatial distribution of VEG showed remarkable consistency with the spatial patterns of soil moisture (**Supplementary Figure S2**; **Figure 7**). Both indices displayed significantly lower values in the northern reaches compared to southern areas of the main watershed. This spatial congruence between VEG and soil moisture validates the methodological robustness of integrating NPP, LAI and ARVI for spatial vegetation characterization, as it effectively captures the eco-hydrological relationships in the watershed system.

### 3.5 The response of soil moisture to vegetation

To examine the spatial correlations and response mechanisms between SM and VEG, we conducted a high-resolution, pixel-scale Spearman correlation analysis using long-term (2017-2024) VEG and SM data.

**Supplementary Figure S3** reveals that in spring, areas with statistically significant correlations (p< 0.05) between SM and VEG at 0–10 cm and 10–20 cm depths were primarily concentrated in the southern main channel region. The correlation coefficients displayed a distinct spatial pattern, with positive correlations in the south and negative correlations in the north. At 20–30 cm depth, positive correlations prevailed throughout the study area, though the spatial extent of significant correlations was reduced compared to shallower layers.

Summer patterns (**Supplementary Figure S4**) showed notable differences from spring conditions. The significantly correlated areas expanded considerably, with the north-south contrast disappearing and the entire basin exhibiting strong positive correlations. This pattern likely reflects summer vegetation growth and maturity, coupled with enhanced soil moistures.

Autumn spatial correlations closely resembled spring patterns (**Supplementary Figure S5**). However, winter showed markedly reduced significance in spatial correlations across the basin, with no clear spatial differentiation (**Supplementary Figure S6**). This seasonal decline may be attributed to vegetation dormancy in winter, particularly for the deciduous tree species that dominate northern regions.

**Figure 8:**
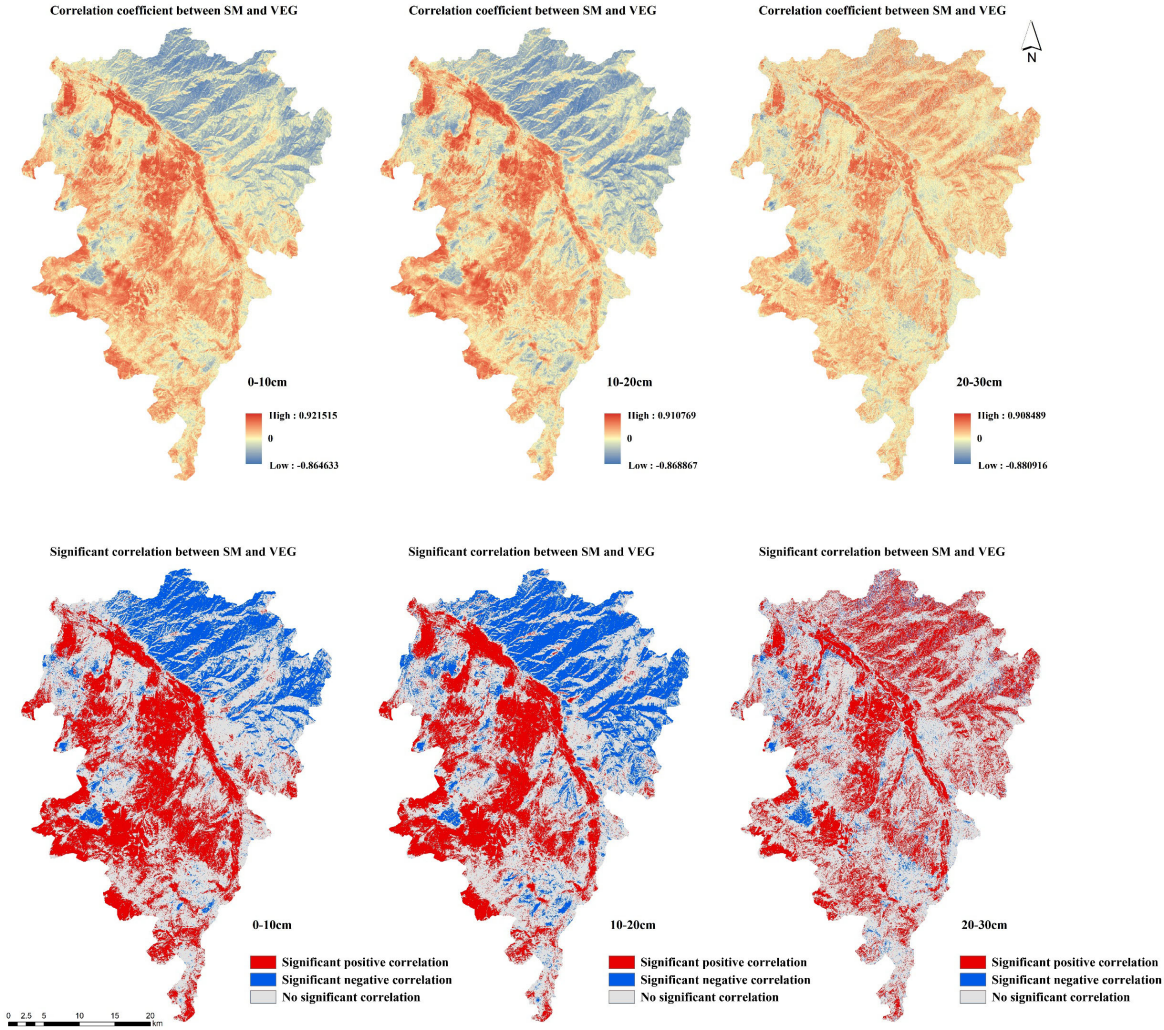
The annual-scale spatial correlation analysis of SM and VEG.

**Figure 8** shows that from 2017 to 2024, soil moistures at all three soil depths exhibited significant correlations with VEG in the basin’s main areas, with significantly correlated areas being much more extensive at the annual scale than at the quarterly scale. Among these, the statistically significant positive and negative correlation areas all exhibit *p*-values< 0.05. At 0–10 cm and 10–20 cm depths, the correlations showed a distinct spatial pattern of significant positive correlations in southern areas and negative correlations in northern regions. In contrast, at 20–30 cm depth, the correlations displayed uniform positive values across the entire study area without the clear north-south differentiation observed in shallower layers.

**Figure 9:**
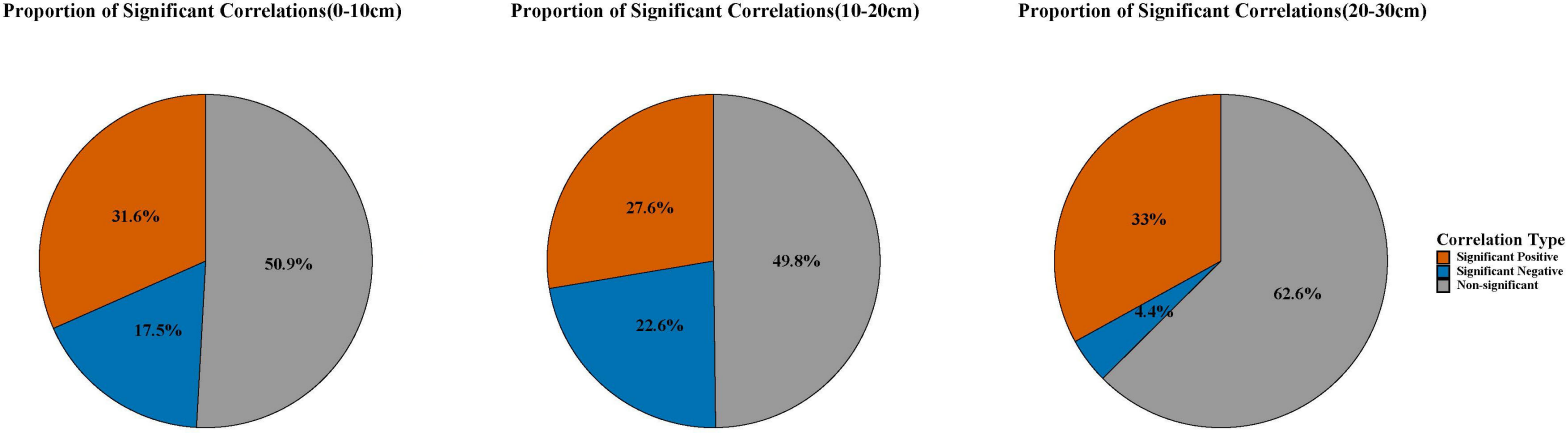
Proportion of significant correlation areas between SM and VEG.

By calculating the proportion of statistically significant correlation areas (**Figure 9**), it can be observed that approximately half of the Wanchuan River Basin shows significant correlations between vegetation and soil moisture at depths of 0–10 cm and 10–20 cm, indicating a strong interactive response between these two variables. In contrast, at the 20–30 cm depth, non-significant correlation areas dominate the basin. As for the significant correlation areas, positive correlation is predominant, suggesting a synergistic relationship between vegetation and soil moisture in the Wanchuan River Basin, where both tend to increase or decrease simultaneously. Moreover, this coordinated pattern is unlikely to be attributed to random chance, as the correlations are statistically significant.

### 3.6 Coupling and coordination analysis of soil moisture and comprehensive vegetation index

The spatial coupling coordination degree between SM and VEG reveals their landscape-scale synergistic interactions (**Supplementary Figure S7**). Summer patterns exhibited pronounced spatial heterogeneity across all three soil depths, consistently demonstrating a clear north-south divergence characterized by coordinated conditions in northern areas and uncoordinated states in southern regions. The Xinglong Mountain area in the southwest stood out with moderate-to-high coordination levels, reflecting strong system synergy where SM and VEG functioned as an integrated organic system with robust sustainable development capacity.

During spring and autumn, watershed-scale coupling coordination primarily displayed marginally coordinated patterns, indicating basic but suboptimal synergistic relationships between systems that would require structural optimization for improved performance.Winter conditions presented generally uncoordinated states prevailing across all depths except the 10-20cm layer. This pattern reflects limited system integration where one component’s development substantially lagged behind the other, particularly in surface and deep soil layers.

**Figure 10:**
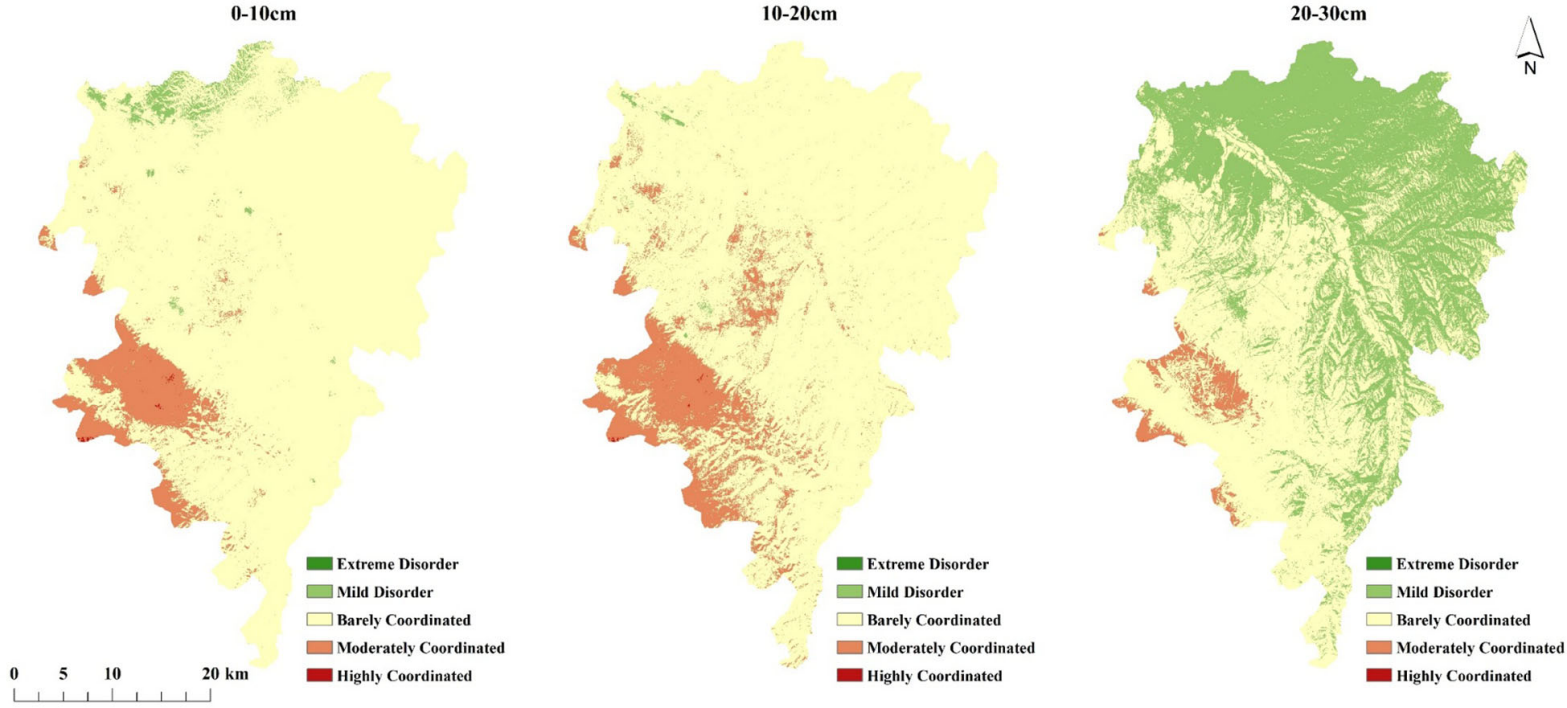
The annual-scale spatial coupling coordination degree of SM and VEG.

**Figure 11:**
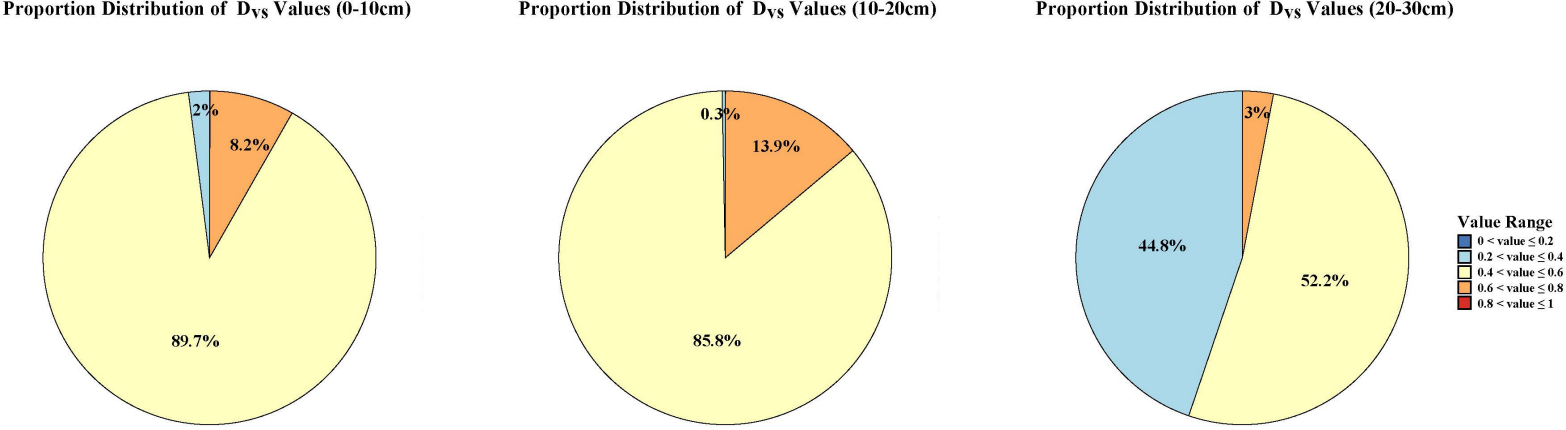
Proportion of coupling coordination degree areas between SM and VEG.

The annual-scale coupling coordination pattern between SM and VEG in the Wanchuan River Basin shows characteristics similar to spring and autumn conditions (**Figure 10**). The proportional distribution of the five coupling coordination degree (D_vs_) levels (**Figure 11**) shows that at the 0–10 cm and 10–20 cm depths, the marginally coordinated category is significantly dominant, accounting for over 85% of the basin. At the 20–30 cm depth, although the marginally coordinated type remains relatively high, its proportion decreases by nearly 30% compared to the shallower depths, while the slightly disordered category increases notably. Overall, marginal coordination represents the primary manifestation of the soil moisture–vegetation coupling relationship in the Wanchuan River Basin.

### 3.7 Analysis of influencing factor importance for D_vs_ indicators

**Figure 12:**
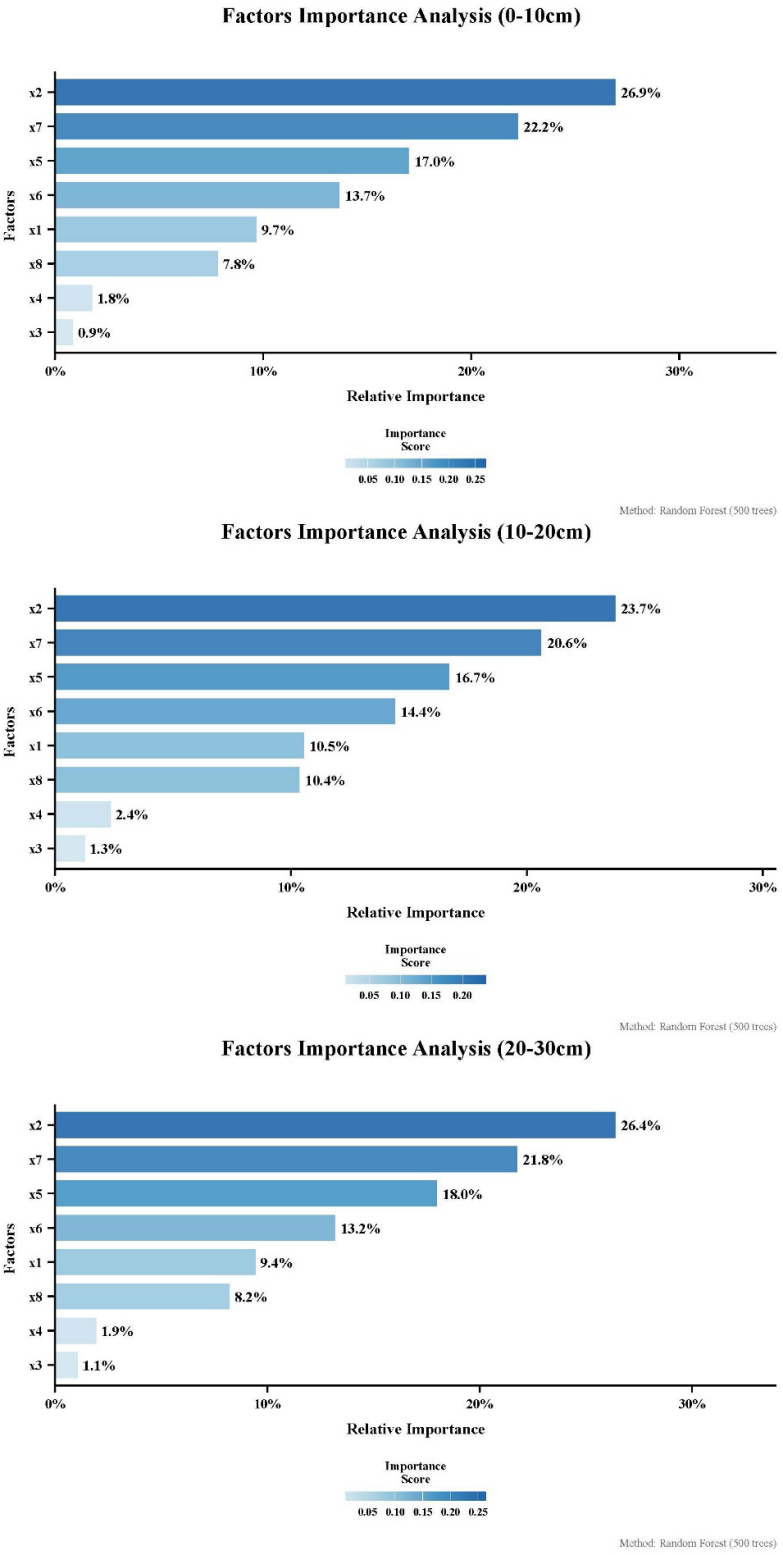
Ranking of influencing factors by importance for D_vs_.

The Gini-index-enhanced random forest algorithm was employed to screen the influencing factors of D_vs_, incorporating factors across four categories: anthropogenic (population density), climatic (mean annual precipitation, mean annual temperature, land surface temperature, potential evapotranspiration), topographic (elevation, slope, aspect). As shown in **Figure 12**, the importance rankings are fully consistent across all three soil depths: land surface temperature (x2) and potential evapotranspiration (x7) are the most significant factors influencing the D_vs_ degree in the Wanchuan River Basin, with their combined contribution close to 50%. Aspect and slope (x3, x4) have the least influence, while elevation (x8) and population density (x1) exhibit similar impact levels. Precipitation (x6) and air temperature (x5) rank below x2 and x7 in importance, though their effects are notably less pronounced than those of land surface temperature and potential evapotranspiration.

## 4 Discussion

To visualize soil moisture and vegetation characteristics and investigate their interactions, this study first employed Pearson correlation analysis to identify indicators significantly correlated with SM for regression model construction, thereby improving model accuracy and enabling multi-temporal spatial visualization of SM. Simultaneously, by evaluating seven commonly used vegetation indices, three vegetation descriptors—vegetation indices, NPP, and LAI—were integrated to develop a comprehensive vegetation index (VEG) equation, facilitating multi-temporal spatial simulation of vegetation conditions.

Pixel-based high-resolution spatial correlation analysis was applied to the generated SM and VEG raster data to preliminarily assess their interactions, including the proportion of statistically significant correlations and their spatial distribution. To gain deeper insight into their synergistic mechanisms, a coupling coordination degree model was adopted to quantify the coordination relationship, clarifying the synergistic patterns and spatial structure between the two variables.

Furthermore, using a Gini index-optimized random forest algorithm, factors directly related to both SM and VEG—including human activity, climatic, and topographic variables—were analyzed to identify the most influential drivers. This analysis provides a scientific basis for offering effective recommendations for the coordinated development of soil moisture and vegetation in the Wanchuan River Basin.

### 4.1 The spatio-temporal variations of the SM and VEG of the Wanchuan River

Soil physicochemical properties serve as crucial indicators for determining soil moisture content. Research by Nguemezi et al. (2020) demonstrated that these properties directly influence soil moisture, thereby limiting plant growth. In this study, with soil moisture content as the dependent variable and SM-related indicators at different soil depths as independent variables, we established regression models for SM at various soil depths to investigate its spatial distribution characteristics. The results indicate that among the physicochemical properties, bulk density and soil porosity exhibit the highest correlation coefficients with SM and appear most frequently as independent variables in the regression models. These two factors are therefore identified as the dominant drivers influencing SM estimation and have the greatest impact on SM variation. This finding aligns with existing studies (Ilek et al., 2017; Cui et al., 2023; Tian et al., 2018), confirming that the water storage capacity of soil layers is closely related to bulk density, and that changes in bulk density affect soil hydraulic properties. The simulation results showed distinct methodological advantages: SMLR proved more suitable for soil moisture inversion at 0-10cm and 20-30cm depths, while ridge regression emerged as the optimal approach for 10-20cm depth soil moisture inversion.These results are consistent with the findings of Alsaleh et al. (2025). SM exhibits no significant variation with depth, showing consistent spatial patterns across all three measured depths: lower values in the northern bank and higher values in the southern bank of the basin. Three interacting mechanisms may explain this spatial heterogeneity: (1) dense vegetation cover and thick litter layers enhance surface soil moisture, (2) reduced potential evapotranspiration decreases moisture loss from deep soil layers, and (3) extensive root systems of mature trees improve water storage capacity. These results demonstrate vegetation’s critical role in regulating complex vertical water redistribution dynamics in forest soil ecosystems.

The establishment of a vegetation status model for the Wanchuan River Basin enables timely and accurate recording of the spatiotemporal patterns and dynamic changes of vegetation cover, providing a scientific basis for strengthening ecological protection and soil-water conservation efforts (Liu D. et al., 2022). For comprehensive vegetation evaluation, principal component analysis was used to study the correlations among three vegetation parameters. The results showed: the absolute magnitude of coefficients in the first principal component equation followed LAI > NPP > ARVI, while ARVI dominated in the second principal component, differing from the results of Zhou et al. (2021). In contrast to Zhou’s study, which combined NDVI, LAI, and NPP for PCA analysis based on subjective judgment without conducting KMO and Bartlett’s tests or correlation analysis, this study implemented a rigorous preliminary screening process. We determined that ARVI, NPP, and LAI are more suitable for constructing a composite vegetation index. The normalized results demonstrate improved accuracy in vegetation characterization. Accordingly, we recommend using a composite vegetation index integrating NPP, LAI, and ARVI for vegetation characterization and its correlation analysis with soil moisture.This conclusion differs from Javier et al. (2025), who identified NDVI as the most suitable vegetation index for moisture-related studies. The discrepancy may be attributed to the high proportion of built-up areas in the Wanchuan River Basin, particularly the main urban area of Yuzhong County, Lanzhou, located on the south bank of the river, where aerosol content is notably elevated. Since ARVI is more effective than NDVI in regions with high aerosol concentrations, these findings possess both theoretical foundation and practical value, making them more suitable for guiding vegetation-soil moisture conservation strategies in the Wanchuan River Basin.

Furthermore, the study showed that temporally, the comprehensive vegetation index exhibited a gradually increasing trend with a clear normal distribution, indicating continuous improvement in vegetation conditions in the study area. High-value areas were mainly distributed in the Xinglong Mountain area in the southwest of the study area, while low-value areas were mainly located in low-coverage regions along the northern bank. The comprehensive vegetation index had significant effects on SM across different periods, consistent with Li et al. (2025)’s research.

### 4.2 Investigating SM–VEG relationships and influencing factors in the Wanchuan River Basin

This study conducted spatial response research between vegetation and soil moisture at a high resolution of 10m×10m, improving analytical precision in spatial relationships and detail processing compared to previous low-resolution grid studies, reflecting that even small vegetation patches have significant effects on soil moisture, similar to Xu et al. (2025)’s findings. However, this study reveals that as soil depth increases, the proportion of statistically significant correlation areas decreases, the synergistic effect between vegetation and soil moisture weakens, and their mutual influence diminishes. This finding contradicts the results reported by Xu et al. (2025). This difference may be due to significant terrain variations in the Wanchuan River Basin, where soils are mainly loose loess, and the dominant vegetation species in most areas are shrubs or herbs that have greater water retention effects on surface soil than deeper layers.

Vegetation-soil coordination is key to the success of farmland-to-forest conversion projects (Feng et al., 2023). Coupling coordination analysis can reflect interactions between vegetation-soil systems and quantitatively evaluate their coordination during ecological development. The vegetation-soil coupling model proposed by Feng et al. (2023) was used to study the synergistic effects between vegetation and soil and determine suitable vegetation restoration measures for local ecosystems. The results showed that the interaction and coordination between hydrological environment and vegetation varied significantly by season: summer showed the greatest differences with coordinated and uncoordinated areas distributed on the north and south banks respectively; spring and autumn showed mostly marginal coordination across the basin; winter showed mostly uncoordinated conditions except at 10-20cm depth. At the annual scale, the coupling coordination degree predominantly exhibits marginal coordination, consistent with the findings of Chen et al. (2022). Except for the Xinglong Mountain area, which demonstrates moderate coordination, most of the basin is characterized by a marginally coordinated state. Simultaneously, the areal proportion results further confirm that marginal coordination serves as the dominant pattern characterizing the soil moisture–vegetation system in the Wanchuan River Basin. These results indicate that although current ecological restoration efforts have established a fundamental synergy between hydrological processes and vegetation dynamics, further optimization of ecosystem structure is still required. Integrating ecological conservation with economic development will be essential to progressively achieve regional sustainable development.

Li et al. (2014) demonstrated the influence of precipitation on soil moisture through correlation analysis, while Wang et al. (2020) using regression and correlation analysis, identified human activities as the most significant factor affecting vegetation indices. Gao et al. (2025) integrating the Optimal Parameter-based Geographical Detector (OPGD), Multiscale Geographically Weighted Regression (MGWR), and Partial Least Squares Structural Equation Modeling (PLS-SEM), found that topographic indicators contributed most to vegetation index variation. Similarly, Sur et al. (2013) confirmed the role of topography in influencing soil moisture. This study comprehensively considered all potential influencing factors and selected eight drivers encompassing human activities, climate, topography, and potential evapotranspiration for analyzing the importance of factors affecting D_vs_. Unlike previous approaches, we employed a Gini index-enhanced random forest algorithm to identify the most critical factors. The results show that land surface temperature and potential evapotranspiration rate are the most important factors influencing D_vs_ in the Wanchuan River Basin. This finding diverges from previous studies, indicating that updated screening methods and improved factor inclusiveness have influenced the outcome. It also demonstrates that in the Wanchuan River Basin, the dominant factors affecting vegetation-soil moisture interactions are not conventional drivers like precipitation, human activities, or topography, but rather more directly influential factors such as potential evapotranspiration and land surface temperature.

### 4.3 Limitations and prospects

This study presents, for the first time, a comprehensive methodology for constructing an comprehensive vegetation index and analyzing the response relationship between soil moisture and vegetation in the Wanchuan River Basin, a major tributary of the Yellow River. It systematically reveals the spatiotemporal distribution patterns and coupling coordination status of soil moisture and vegetation. Although the influencing factors identified in this study differ from previous research findings, the underlying cause can be attributed to the variability in driving factors and soil physicochemical characteristics across different major tributaries of the Yellow River. This variability necessitates tailored model selection and case-specific analysis.

Furthermore, the foundational data used in this study were derived from Sentinel-1 and Sentinel-2 imagery, which have been operational only since approximately 2016. This significantly limits the long-term applicability of the results. Additionally, there are resolution discrepancies between the high-resolution Sentinel data and other datasets such as NPP, LAI, population density, and climatic factors.

To address these limitations and enhance the generalizability of the research, future work should focus on the following aspects:

1. Conduct systematic field sampling and image processing in other sub-basins of the upper Yellow River, such as the Zuli River Basin closest to the study area, to improve the generalizability of the soil moisture–vegetation coupling coordination model and the integrated vegetation index.
2. Integrate multi-sensor satellite data, including Landsat and MODIS, to develop accurate soil moisture–vegetation coupling coordination models and comprehensive vegetation index construction methods for periods prior to 2016.

## 5 Conclusion

This study systematically developed inversion models for assessing soil moisture in the Wanchuan River Basin by integrating field-measured soil physicochemical properties, Sentinel-1/2 remote sensing data, and multiple vegetation indices through comprehensive regression analysis. The results demonstrate that bulk density and soil porosity constitute the most critical physicochemical factors influencing soil moisture. The comparative evaluation of regression methods demonstrates that ridge regression is more suitable for the intermediate soil layer, while SMLR performs better for moisture inversion in both surface and deep soil layers, showing optimal simulation effectiveness for surface soil moisture in this ecologically sensitive area. No significant vertical variation in soil moisture was observed across the study area, while the overall spatial distribution exhibited a clear north–south gradient, with lower values in the northern part and higher values in the southern part.

Principal component analysis incorporating NPP, LAI, and multiple vegetation indices identified ARVI as the most suitable vegetation index to combine with NPP and LAI for constructing a comprehensive vegetation index (VEG). Temporal analysis of VEG revealed a gradually increasing trend with normal distribution characteristics, indicating measurable improvements in vegetation conditions across the basin. High-resolution spatial correlation analysis reveals strong interactions between soil moisture and vegetation, with statistically significant positive correlations dominating among the significant areas. Spatially, a consistent south-positive/north-negative correlation pattern is observed, highlighting the remarkable mutual influence between soil moisture and vegetation dynamics.

Analysis using the coupling coordination degree model revealed distinct seasonal patterns in SM-VEG relationships, with summer showing pronounced spatial variability featuring coordinated conditions in southern areas and uncoordinated states in northern sectors, while spring and autumn exhibited widespread marginal coordination, and winter displayed predominantly uncoordinated conditions basin-wide. Marginal coordination represents the predominant manifestation of the soil moisture–vegetation coupling relationship throughout the entire river basin. The variable importance screening results indicate that land surface temperature and potential evapotranspiration are the most critical factors influencing the soil moisture–vegetation coupling coordination degree in the Wanchuan River Basin. These findings demonstrate that although ecological restoration efforts on the Loess Plateau have established a marginal synergistic effect between soil moisture and vegetation, the current level of coupling coordination remains suboptimal. Continued commitment to vegetation improvement and soil-water conservation measures is essential to regulate land surface temperature and potential evapotranspiration patterns. Further optimization of ecosystem structure and implementation of integrated management strategies will be crucial for progressively achieving sustainable development goals in the Yellow River Basin.

## Data Availability

The original contributions presented in the study are included in the article/**Supplementary Material**. Further inquiries can be directed to the corresponding author.
