# Supplementary material for: The response and coupling coordination of soil moisture to vegetation in the Yellow River’s primary tributaries: a multi-source data analysis of the Wanchuan River Basin
Source: Front Plant Sci. 2026 Jan 19;16:1700203. doi: 10.3389/fpls.2025.1700203 (PMC12862093; doi:10.3389/fpls.2025.1700203)
Supplement: Supplementary file 1 [file DataSheet1.docx]

**Supplementary information**


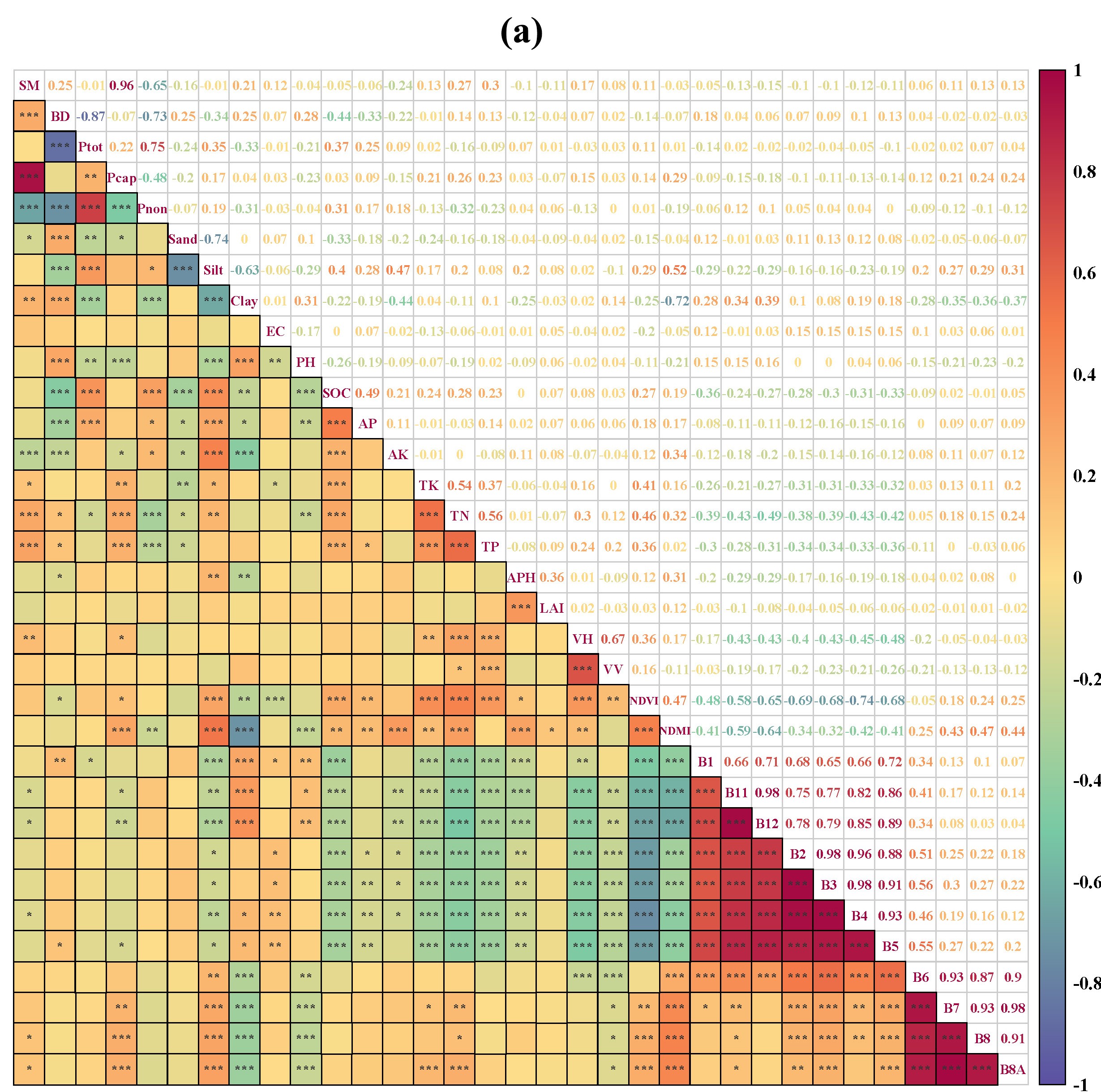


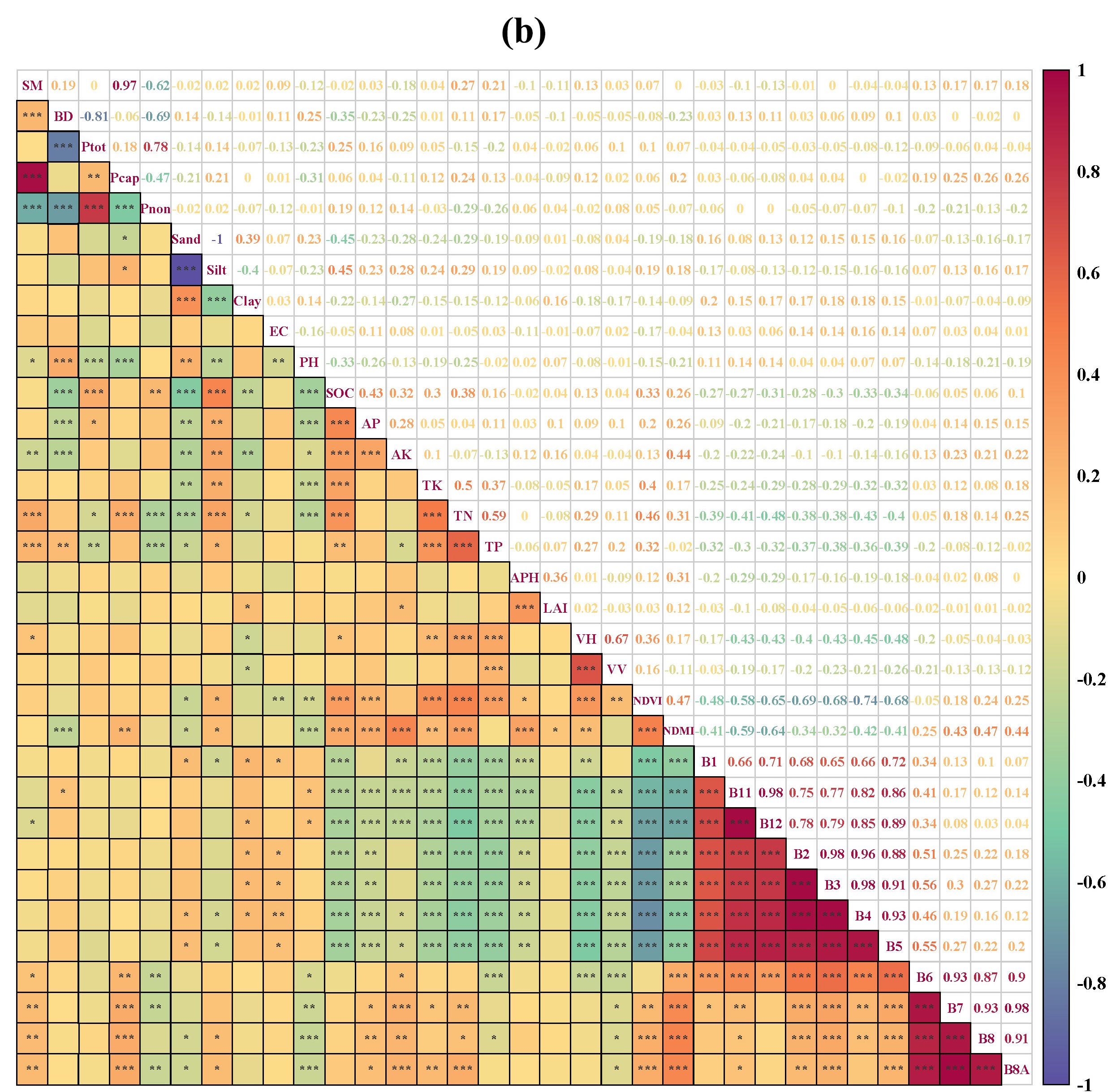


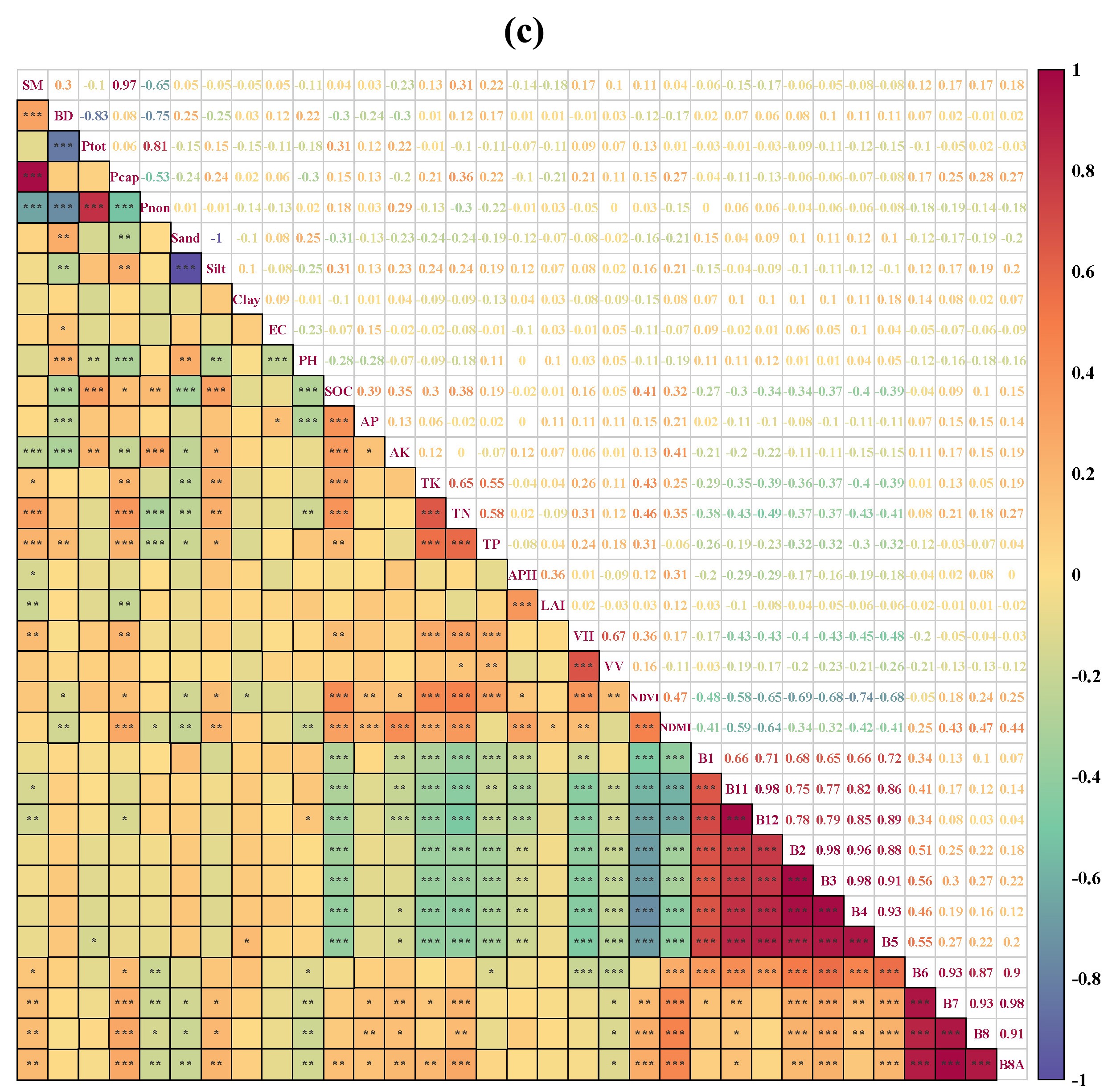


**Fig. S1.** Correlation between soil moisture at three distinct depths and indicators of soil moisture:(a)0-10cm; (b)10-20cm; (c)20-30cm (*****: *p* < 0.05; ******: *p* < 0.01; *******: *p* < 0.01)


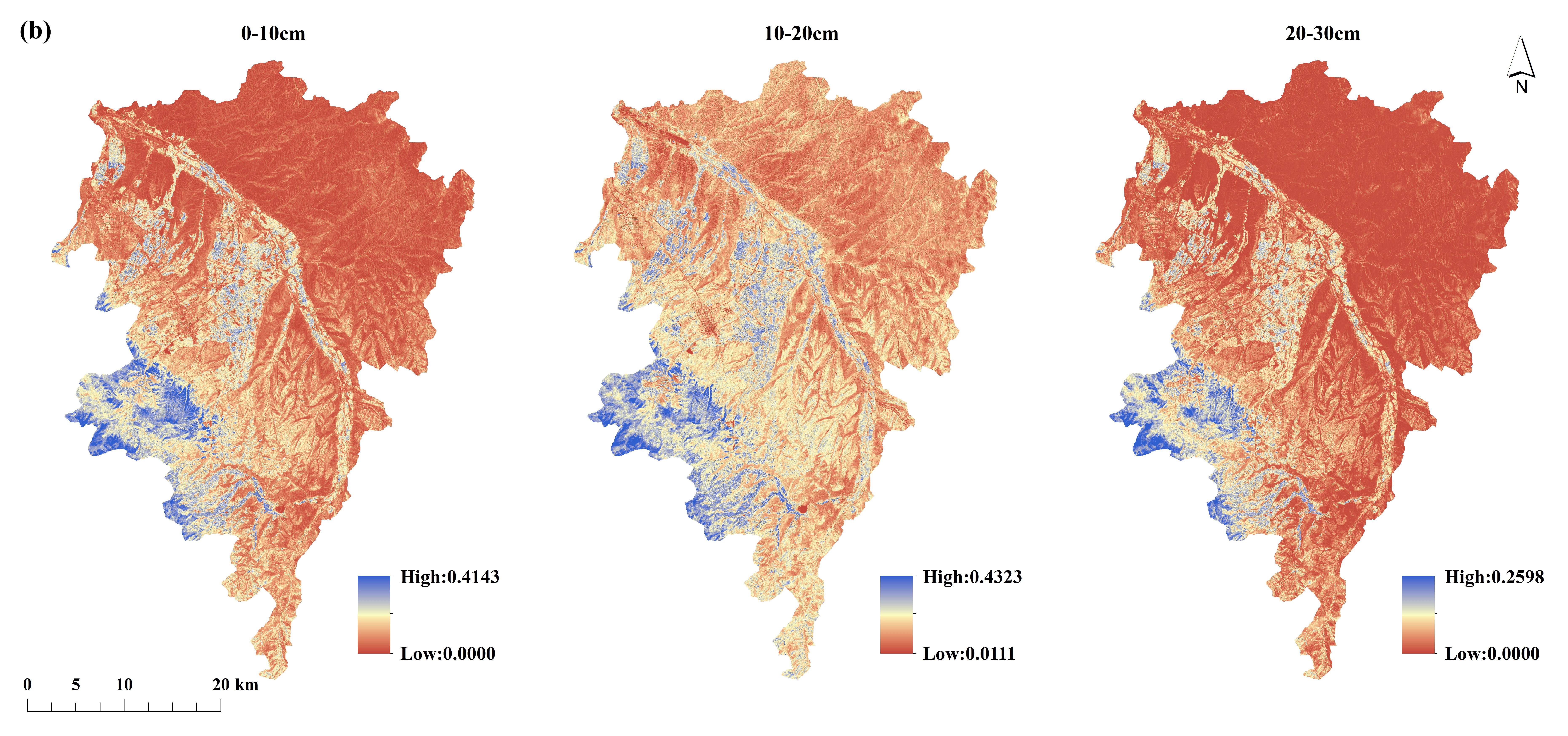

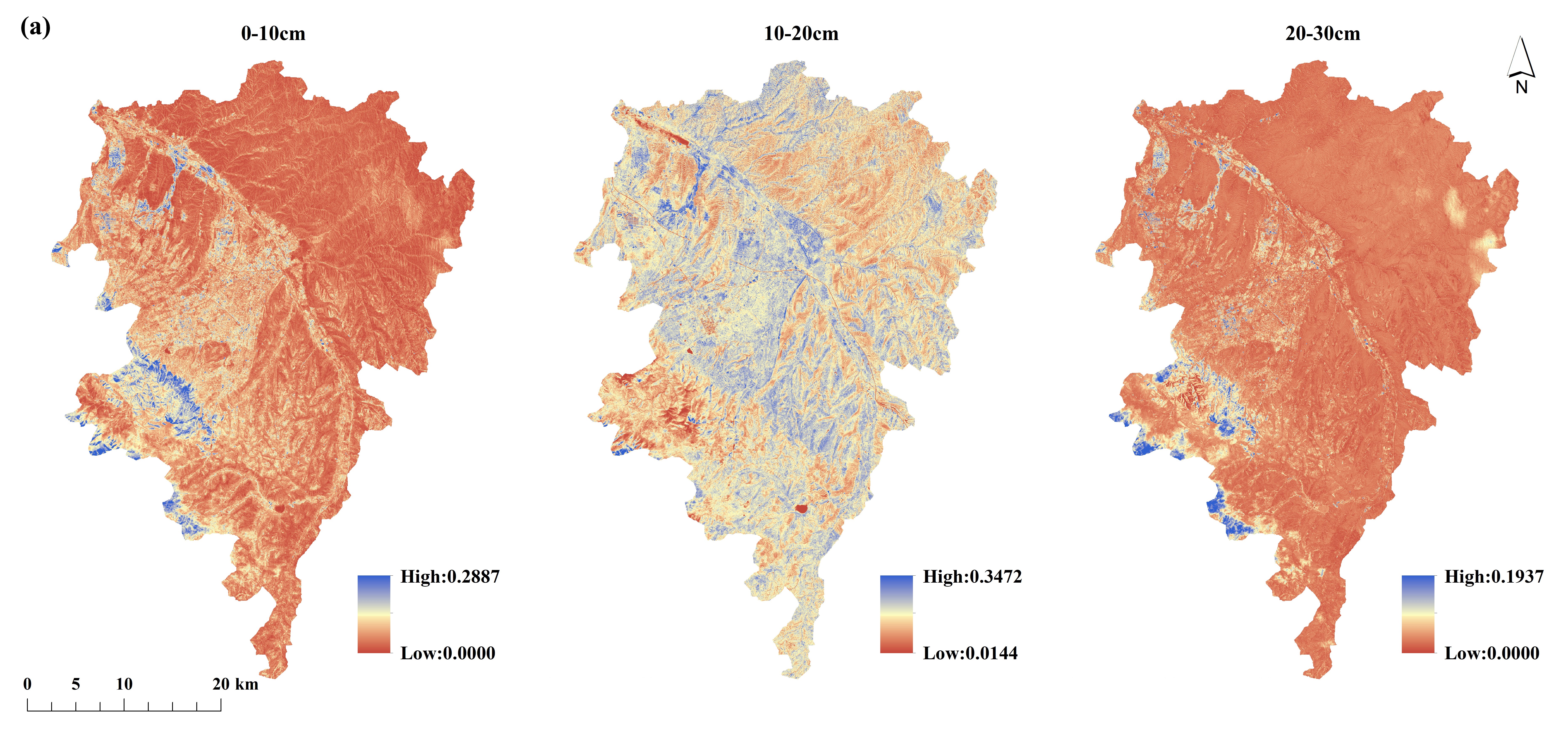


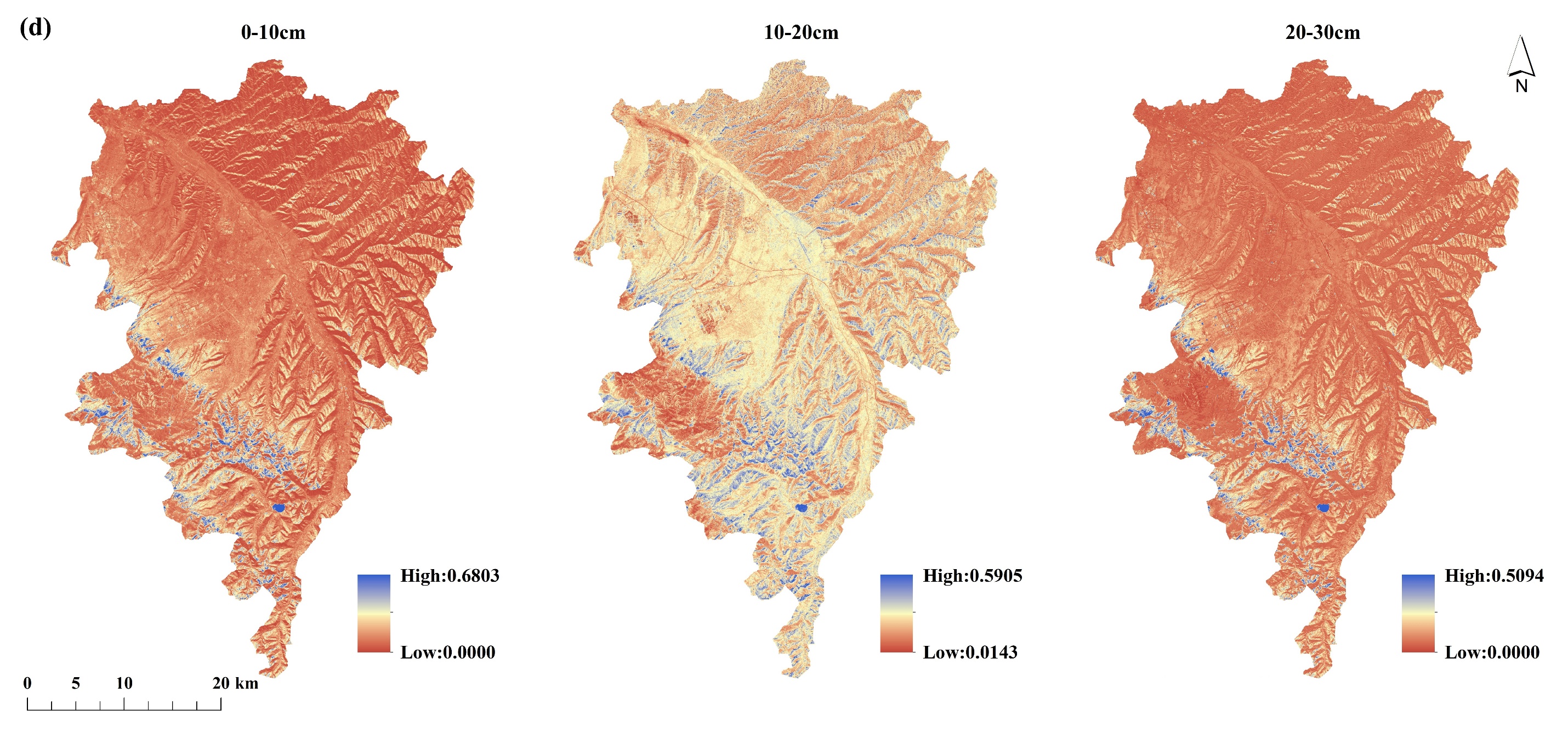

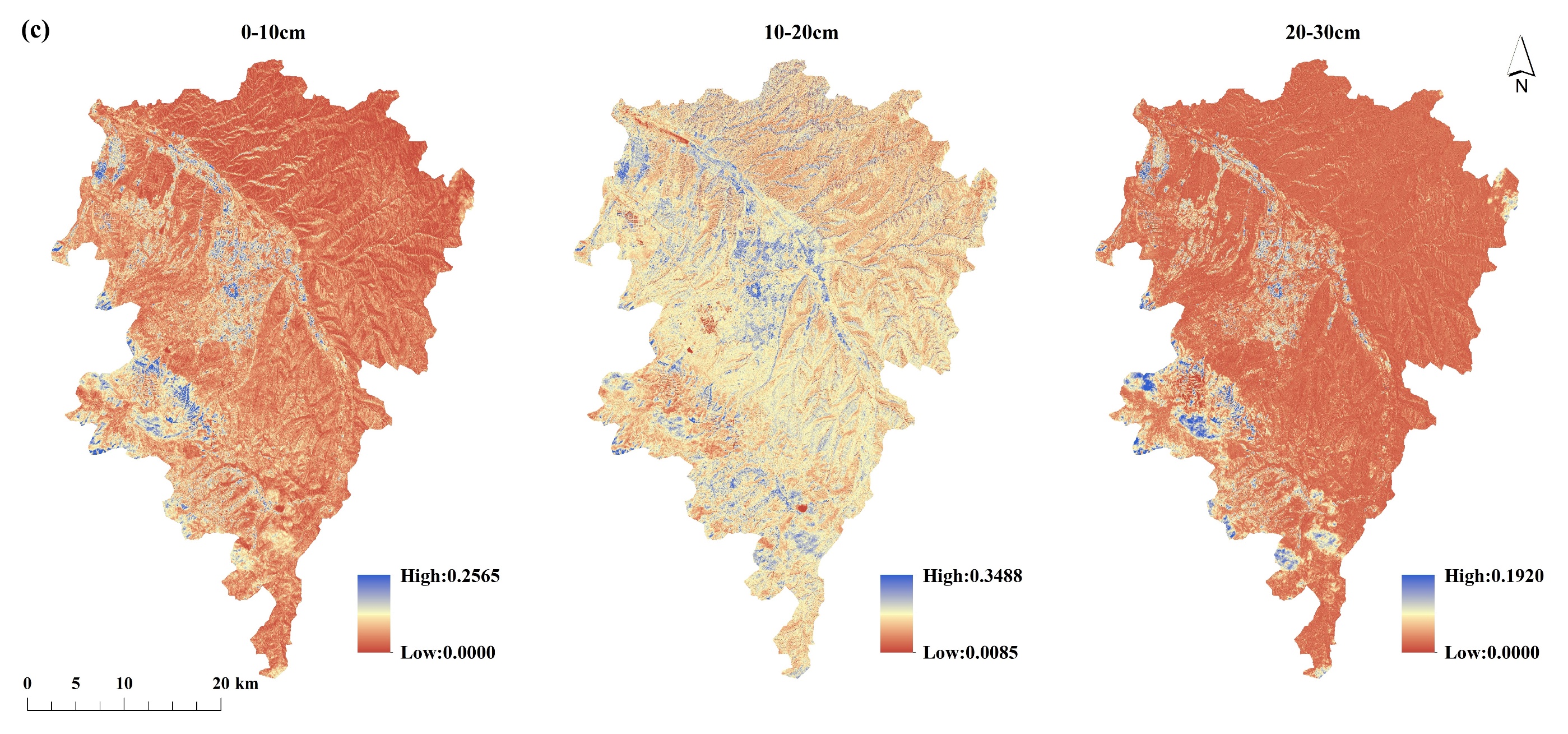


**Fig. S2.** The Seasonal-Scale Spatial distribution of SM from 2017 to 2024:(a) Spring;(b) Summer;(c) Autumn;(d) Winter

**Table S1**

Correlation Matrix of Vegetation Factors

| Correlation Matrix | Vegetation factors | Zscore: NPP | Zscore: LAI | Zscore: NDVI |
| --- | --- | --- | --- | --- |
| Correlation | Zscore: NPP | 1.000 | 0.859 | 0.195 |
|  | Zscore: LAI | 0.859 | 1.000 | 0.101 |
|  | Zscore: NDVI | 0.195 | 0.101 | 1.000 |
| Test of significance | Zscore: NPP |  | 0.000 | 0.000 |
|  | Zscore: LAI | 0.000 |  | 0.000 |
|  | Zscore: NDVI | 0.000 | 0.000 |  |
|  |  | Zscore: NPP | Zscore: LAI | Zscore: RVI |
|  | Zscore: NPP | 1.000 | 0.859 | 0.423 |
| Correlation | Zscore: LAI | 0.859 | 1.000 | 0.403 |
|  | Zscore: RVI | 0.425 | 0.403 | 1.000 |
|  | Zscore: NPP |  | 0.000 | 0.000 |
| Test of significance | Zscore: LAI | 0.000 |  | 0.000 |
|  | Zscore: RVI | 0.000 | 0.000 |  |
|  |  | Zscore: NPP | Zscore: LAI | Zscore: GNDVI |
|  | Zscore: NPP | 1.000 | 0.859 | 0.208 |
| Correlation | Zscore: LAI | 0.859 | 1.000 | 0.156 |
|  | Zscore: GNDVI | 0.208 | 0.156 | 1.000 |
|  | Zscore: NPP |  | 0.000 | 0.000 |
| Test of significance | Zscore: LAI | 0.000 |  | 0.000 |
|  | Zscore: GNDVI | 0.000 | 0.000 |  |
|  |  | Zscore: NPP | Zscore: LAI | Zscore: ARVI |
|  | Zscore: NPP | 1.000 | 0.859 | 0.606 |
| Correlation | Zscore: LAI | 0.859 | 1.000 | 0.530 |
|  | Zscore: ARVI | 0.606 | 0.530 | 1.000 |
|  | Zscore: NPP |  | 0.000 | 0.000 |
| Test of significance | Zscore: LAI | 0.000 |  | 0.000 |
|  | Zscore: ARVI | 0.000 | 0.000 |  |
|  |  | Zscore: NPP | Zscore: LAI | Zscore: DVI |
|  | Zscore: NPP | 1.000 | 0.859 | 0.366 |
| Correlation | Zscore: LAI | 0.859 | 1.000 | 0.301 |
|  | Zscore: DVI | 0.366 | 0.301 | 1.000 |
|  | Zscore: NPP |  | 0.000 | 0.000 |
| Test of significance | Zscore: LAI | 0.000 |  | 0.000 |
|  | Zscore: DVI | 0.000 | 0.000 |  |
|  |  | Zscore: NPP | Zscore: LAI | Zscore: EVI |
|  | Zscore: NPP | 1.000 | 0.859 | 0.073 |
| Correlation | Zscore: LAI | 0.859 | 1.000 | 0.016 |
|  | Zscore: EVI | 0.073 | 0.016 | 1.000 |
|  | Zscore: NPP |  | 0.000 | 0.000 |
| Test of significance | Zscore: LAI | 0.000 |  | 0.000 |
|  | Zscore: EVI | 0.000 | 0.000 |  |
|  |  | Zscore: NPP | Zscore: LAI | Zscore: SAVI |
|  | Zscore: NPP | 1.000 | 0.859 | 0.389 |
| Correlation | Zscore: LAI | 0.859 | 1.000 | 0.336 |
|  | Zscore: SAVI | 0.389 | 0.336 | 1.000 |
|  | Zscore: NPP |  | 0.000 | 0.000 |
| Test of significance | Zscore: LAI | 0.000 |  | 0.000 |
|  | Zscore: SAVI | 0.000 | 0.000 |  |

**Table S2**

KMO and Bartlett's Test of Vegetation factors

| Kaiser-Meyer-Olkin Measure of Sampling Adequacy | | 0.495 |
| --- | --- | --- |
| Bartlett’s Test of Sphericity | Approximate Chi-squared value | 1281567.644 |
|  | degree of freedom | 3 |
|  | significance | 0.000 |

1. NDVI

| Kaiser-Meyer-Olkin Measure of Sampling Adequacy | | 0.598 |
| --- | --- | --- |
| Bartlett’s Test of Sphericity | Approximate Chi-squared value | 1417283.312 |
|  | degree of freedom | 3 |
|  | significance | 0.000 |

1. RVI

| Kaiser-Meyer-Olkin Measure of Sampling Adequacy | | 0.516 |
| --- | --- | --- |
| Bartlett’s Test of Sphericity | Approximate Chi-squared value | 1272486.253 |
|  | degree of freedom | 3 |
|  | significance | 0.000 |

1. GNDVI

| Kaiser-Meyer-Olkin Measure of Sampling Adequacy | | 0.652 |
| --- | --- | --- |
| Bartlett’s Test of Sphericity | Approximate Chi-squared value | 1606298.215 |
|  | degree of freedom | 3 |
|  | significance | 0.000 |

1. ARVI

| Kaiser-Meyer-Olkin Measure of Sampling Adequacy | | 0.558 |
| --- | --- | --- |
| Bartlett’s Test of Sphericity | Approximate Chi-squared value | 1362776.033 |
|  | degree of freedom | 3 |
|  | significance | 0.000 |

1. DVI

| Kaiser-Meyer-Olkin Measure of Sampling Adequacy | | 0.483 |
| --- | --- | --- |
| Bartlett’s Test of Sphericity | Approximate Chi-squared value | 1256927.792 |
|  | degree of freedom | 3 |
|  | significance | 0.000 |

1. EVI

| Kaiser-Meyer-Olkin Measure of Sampling Adequacy | | 0.573 |
| --- | --- | --- |
| Bartlett’s Test of Sphericity | Approximate Chi-squared value | 1380729.488 |
|  | degree of freedom | 3 |
|  | significance | 0.000 |

(g) SAVI


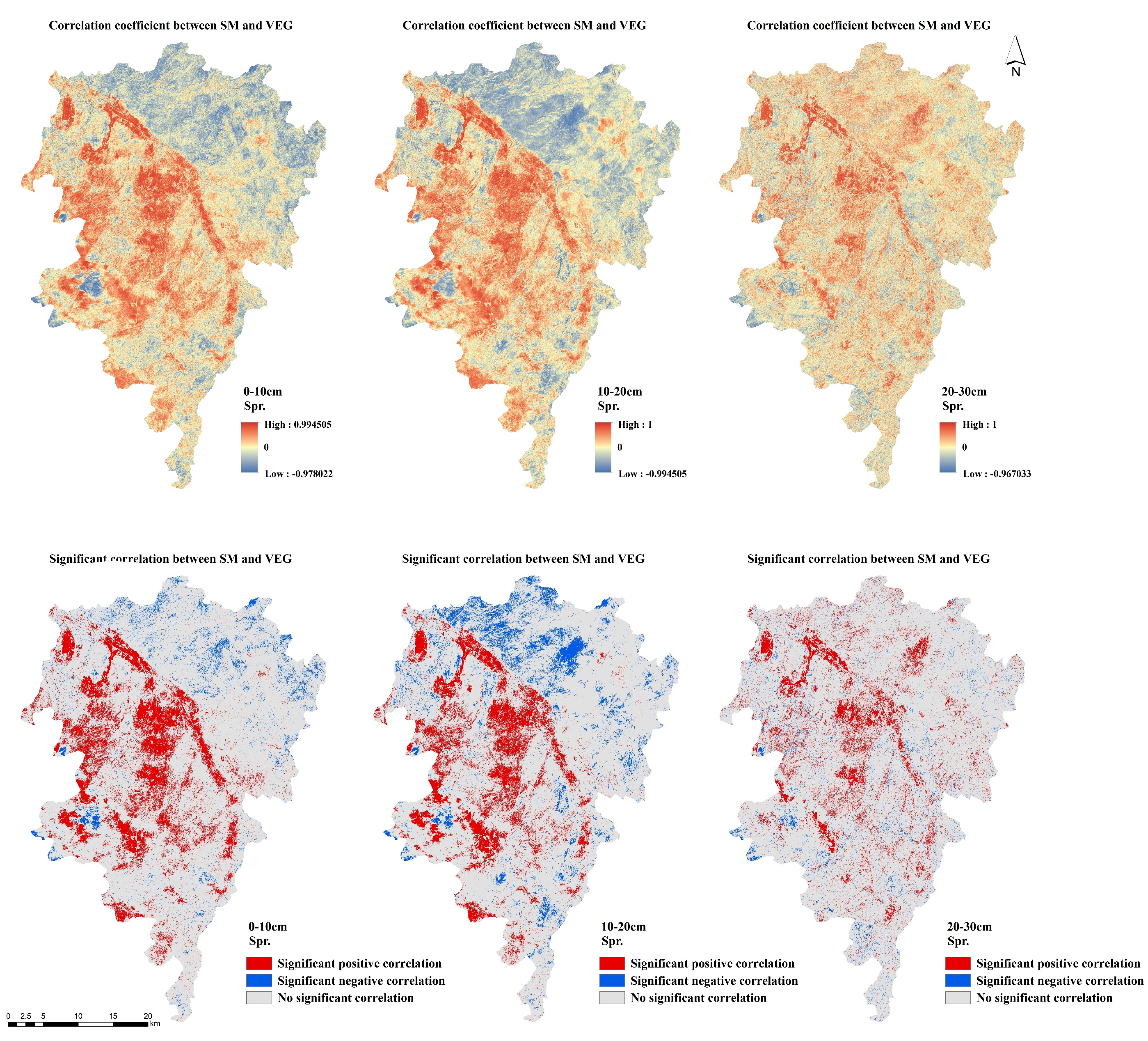


**Fig. S3.** Spring spatial correlation analysis of SM and VEG


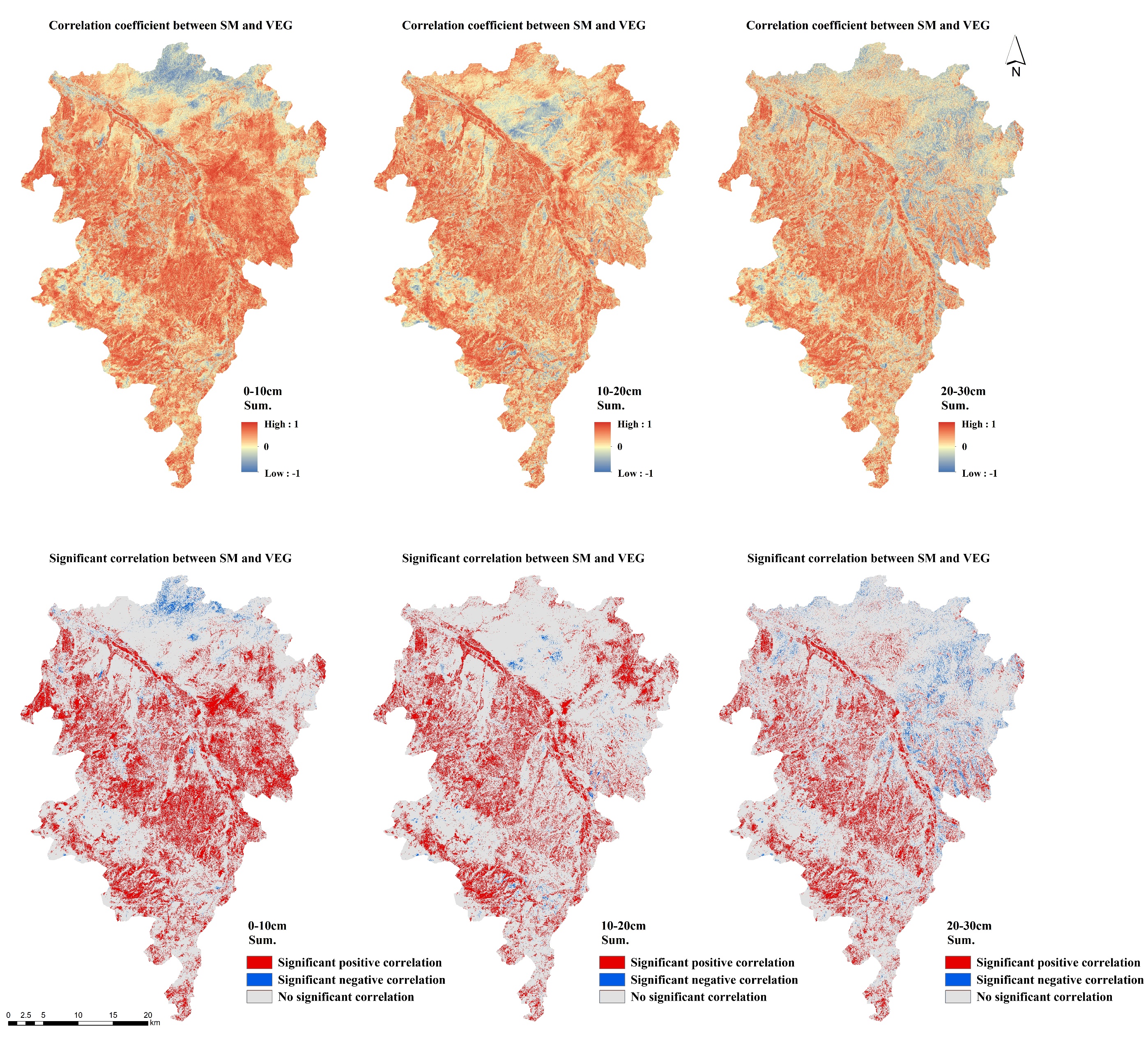


**Fig. S4.** Summer spatial correlation analysis of SM and VEG


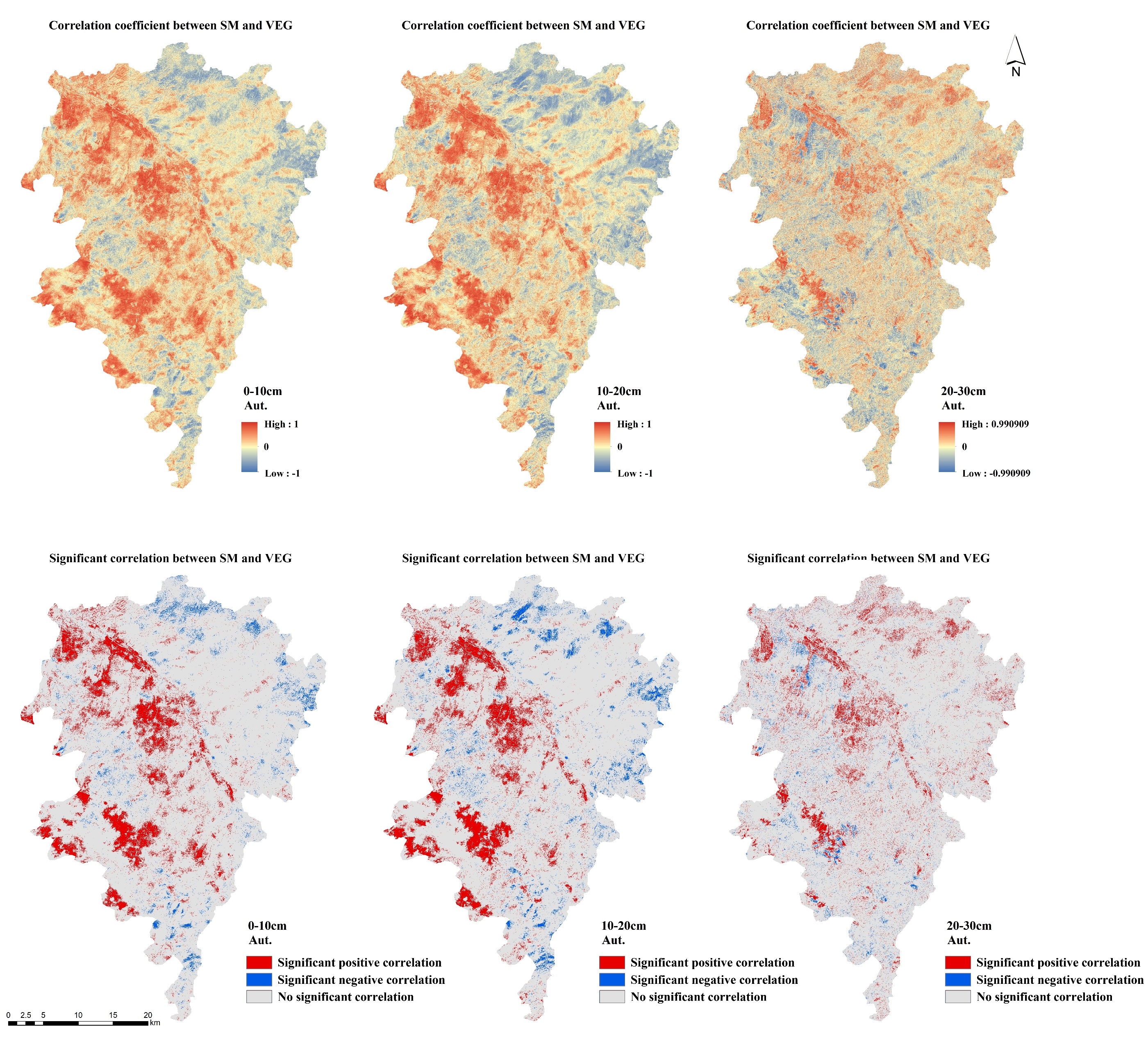


**Fig. S5.** Autumn spatial correlation analysis of SM and VEG


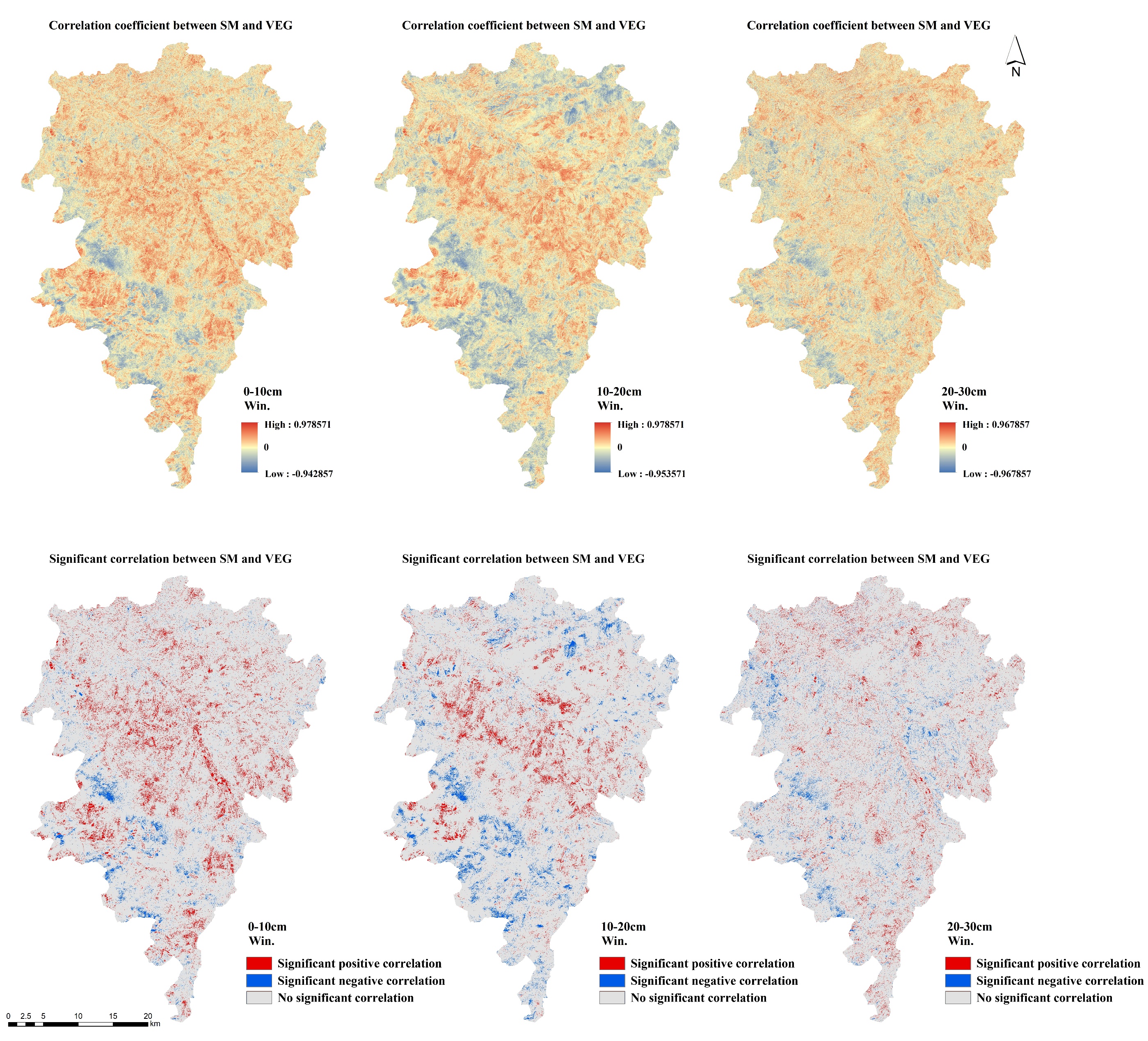


**Fig. S6.** Winter spatial correlation analysis of SM and VEG


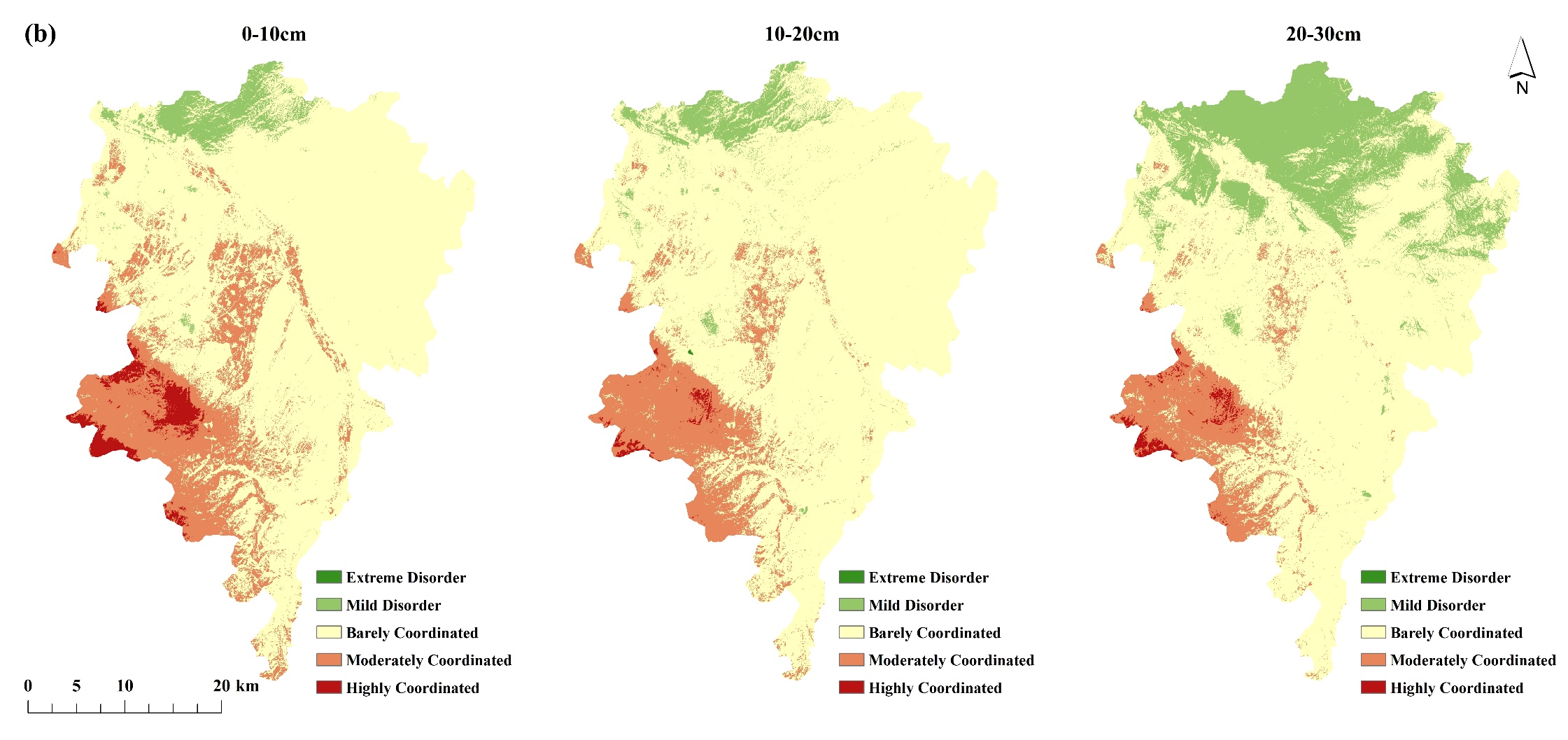

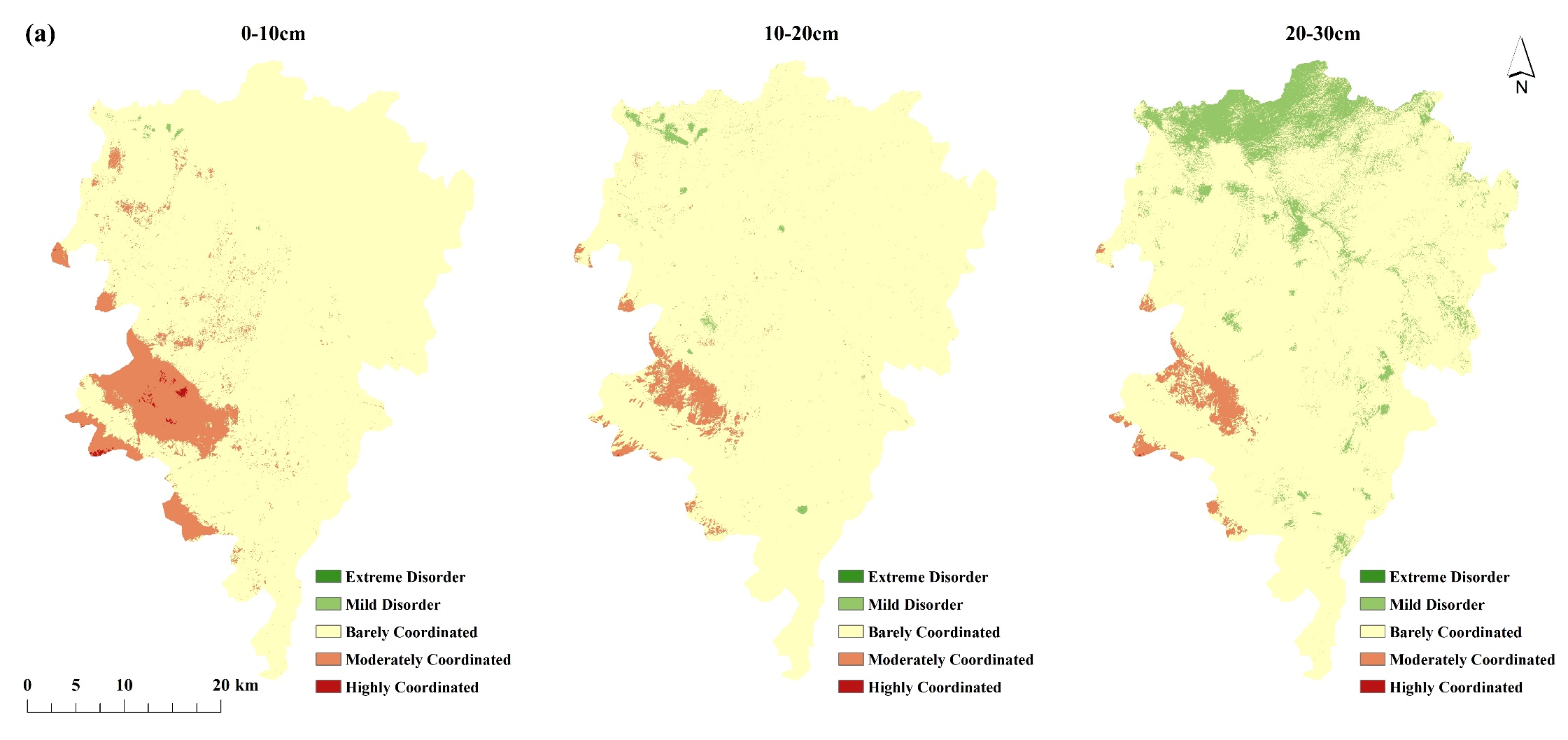


**
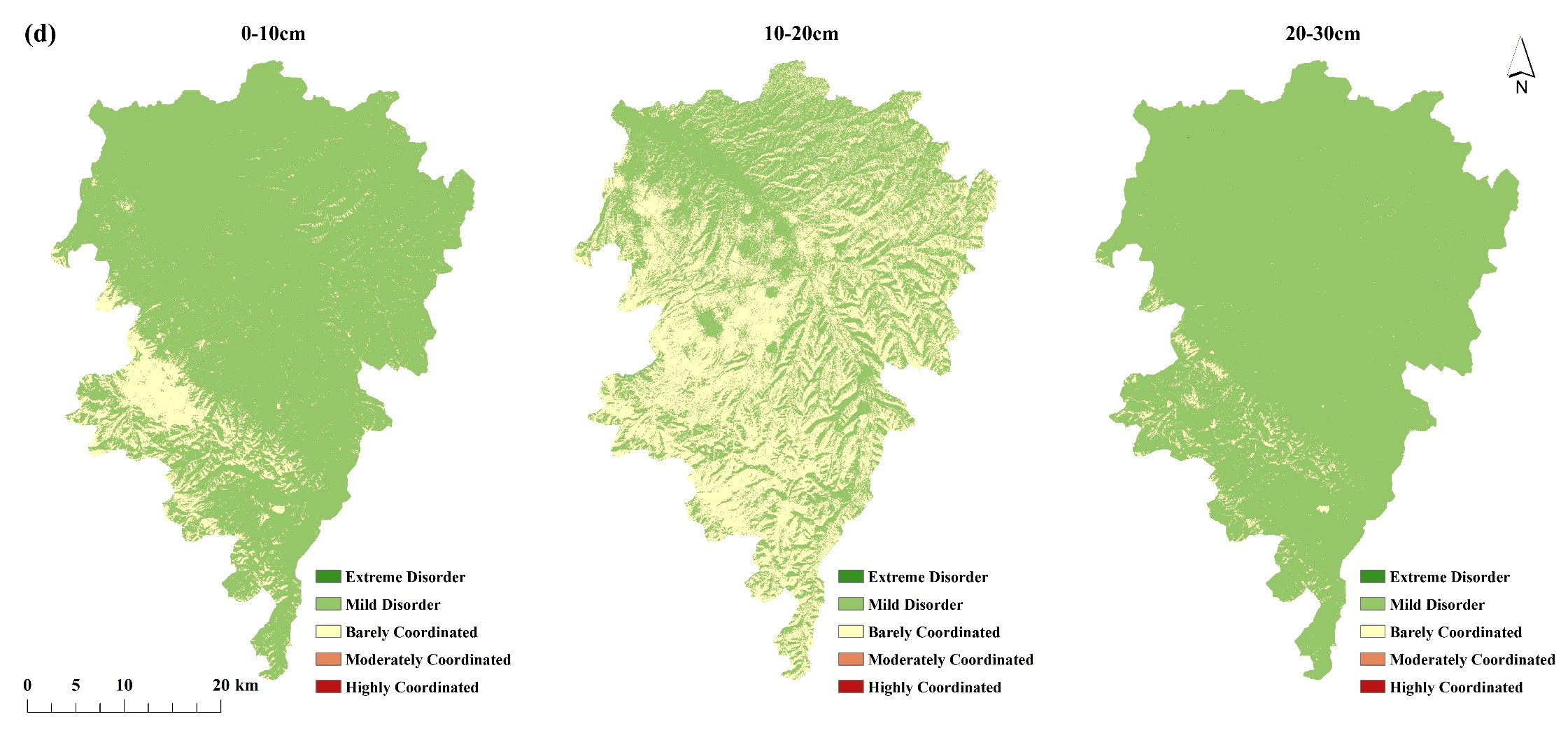

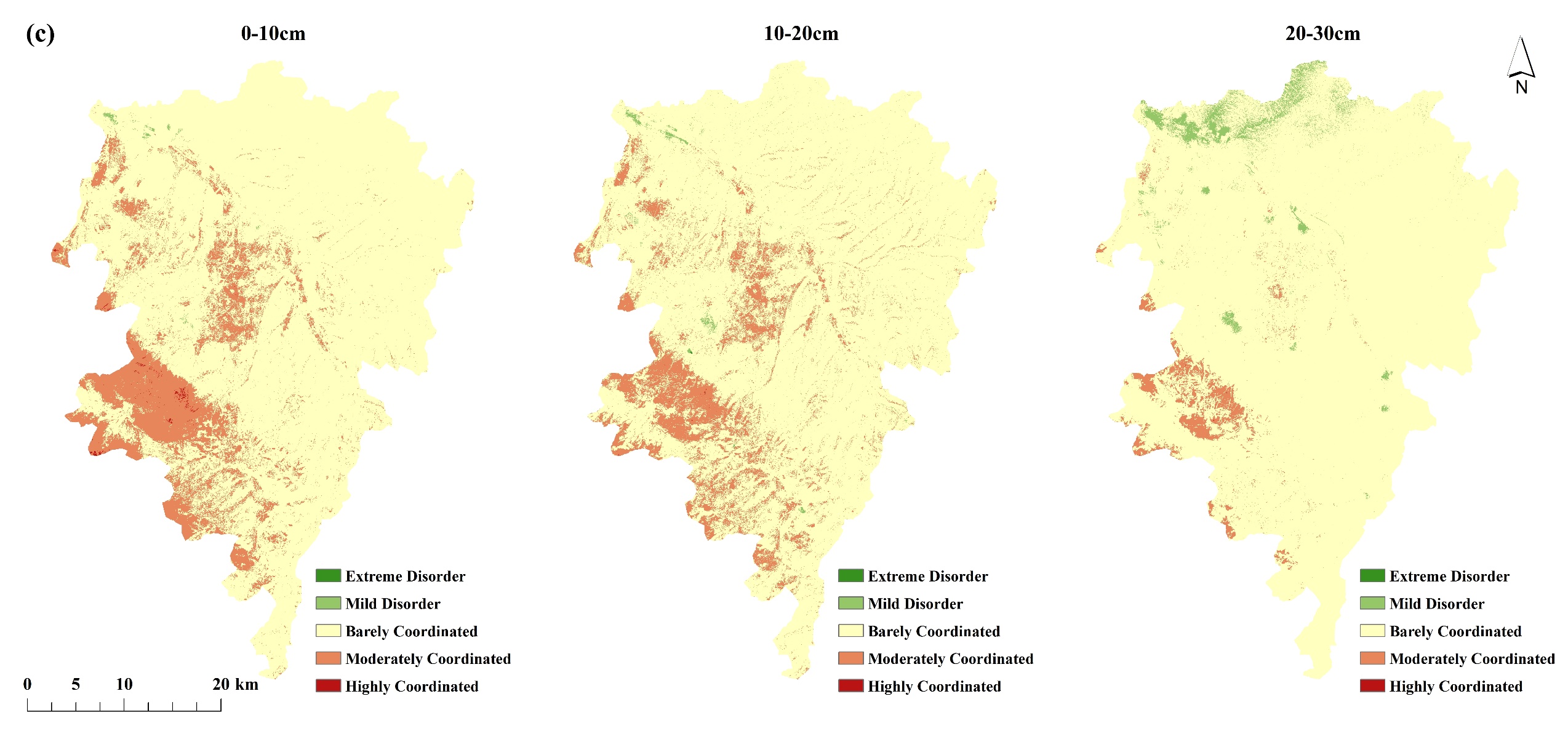
**

**Fig. S7.** The Seasonal-Scale Spatial Coupling Coordination Degree of SM and VEG : (a) Spring;(b) Summer;(c) Autumn;(d) Winter
